# Building and identifying highly active oxygenated groups in carbon materials for oxygen reduction to H_2_O_2_

**DOI:** 10.1038/s41467-020-15782-z

**Published:** 2020-05-05

**Authors:** Gao-Feng Han, Feng Li, Wei Zou, Mohammadreza Karamad, Jong-Pil Jeon, Seong-Wook Kim, Seok-Jin Kim, Yunfei Bu, Zhengping Fu, Yalin Lu, Samira Siahrostami, Jong-Beom Baek

**Affiliations:** 1 School of Energy and Chemical Engineering/Center for Dimension-Controllable Organic Frameworks, Ulsan National Institute of Science and Technology (UNIST), Ulsan, 44919 South Korea; 2 CAS Key Laboratory of Materials for Energy Conversion, Department of Materials Science and Engineering, University of Science and Technology of China (USTC), 230026 Hefei, P. R. China; 3 Department of Chemical and Petroleum Engineering, University of Calgary, 2500 University Drive NW, Calgary, AB T2N 1N4 Canada; 4 Jiangsu Key Laboratory of Atmospheric Environment Monitoring and Pollution Control, School of Environmental Science and Engineering, Nanjing University of Information Science and Technology (NUIST), 219 Ningliu, 210044 Nanjing, Jiangsu P. R. China; 5 Synergetic Innovation Center of Quantum Information and Quantum Physics and Hefei National Laboratory for Physical Sciences at Microscale, University of Science and Technology of China (USTC), 230026 Hefei, P. R. China; 6 Department of Chemistry, University of Calgary, 2500 University Drive NW, Calgary, AB T2N 1N4 Canada

**Keywords:** Density functional theory, Electrocatalysis, Synthesis and processing

## Abstract

The one-step electrochemical synthesis of H_2_O_2_ is an on-site method that reduces dependence on the energy-intensive anthraquinone process. Oxidized carbon materials have proven to be promising catalysts due to their low cost and facile synthetic procedures. However, the nature of the active sites is still controversial, and direct experimental evidence is presently lacking. Here, we activate a carbon material with dangling edge sites and then decorate them with targeted functional groups. We show that quinone-enriched samples exhibit high selectivity and activity with a H_2_O_2_ yield ratio of up to 97.8 % at 0.75 V vs. RHE. Using density functional theory calculations, we identify the activity trends of different possible quinone functional groups in the edge and basal plane of the carbon nanostructure and determine the most active motif. Our findings provide guidelines for designing carbon-based catalysts, which have simultaneous high selectivity and activity for H_2_O_2_ synthesis.

## Abstract (web summary)

The identity of catalytic sites for H_2_O_2_ generation in carbon-based materials remains controversial with limited experimental evidence to date. Here, the authors decorate various target functional groups on carbon materials and quinone-enriched samples exhibit the highest activity and selectivity.

## Introduction

Hydrogen peroxide is widely used as a green oxidant in disinfectants, bleaching agents, sanitizing agents, chemical synthesis, and even as a potential energy carrier^1–14^. According to a report by Global Industry Analysts, Inc.^15^, the global consumption of H_2_O_2_ is projected to reach 6.0 million metric tons by 2024. Approximately 99% of all H_2_O_2_ is currently produced using the multi-step anthraquinone process. However, this process is energy intensive, and can only be performed in a centralized factory^1,5,10^. The inherent complexity of the anthraquinone process has driven many researchers to investigate one-step methods, which can continuously produce H_2_O_2_ on-site at small scale using a simple device^1,5^. Among these, the electrochemical synthesis approach is one of the best choices to meet the above demands.

Metal-free oxidized carbon materials have received particular attention for this application because they offer a simple synthetic procedure, cost-effectiveness, as well as high activity and selectivity^16–29^. They allow the incorporation of a variety of oxygen functional groups, with the potential to widely tune performance, and to optimize active sites. For example, Lu et al. recently used nitric acid to oxidize carbon nanotubes and demonstrated that the resulting oxidized carbon-material was highly active for oxygen reduction to hydrogen peroxide (ORHP)^16^. They considered the active sites to be the carbon atoms adjacent to carboxylic acid and etheric groups (–COOH and C–O–C). Kim et al. used few-layer mildly reduced graphene oxide electrodes, by partially removing oxygen from graphene oxide using hydrothermal heating without a reducing agent, and showed that *sp*^2^-hybridized carbon near ring ethers along the sheet edges were the most active sites for ORHP^17^.

All of these pioneering studies apply harsh oxidation conditions to cleave the strong *sp*^2^ C–C bonds^16,30^ and incorporate oxygen functional groups, and this in turn makes it difficult to functionalize the carbon materials with targeted groups. As a result, the oxygen functional groups inevitably saturate the dangling bonds on the edges with sophisticated multi-components, which hinders systematic experimental study. It becomes inherently difficult to accurately identify the most active functional groups, because the resolution of ordinary characterization methods is not high enough to distinguish between similar groups.

From the industrial anthraquinone process, we know that quinones are the champion catalytically active oxygen functional groups towards H_2_O_2_ synthesis^10,31^. Herein we demonstrate a facile synthesis method to incorporate quinone functional groups in carbon nanostructures. For comparison, we also employ a pre-activated method to build carbonyl-enriched and etheric ring-enriched graphitic nanoplatelets (denoted as GNP_C=O_ and GNP_C–O–C_, respectively). Using fine characterization methods, such as soft XANES, XPS, FTIR, and CV, we show that each of our samples have targeted and desired functional groups (etheric ring, carboxyl and quinone). Electrochemical measurements reveal that the sample with abundant quinone functional group (GNP_C=O,1_) exhibits high selectivity, with a H_2_O_2_ yield ratio of 97.8% at 0.75 V, which is superior to previously reported etheric ring and carboxylic acid groups.

We further demonstrate the activity of the quinone functional groups by examining standalone quinone molecular systems. Finally, we use density functional theory (DFT) calculations to model different possible quinone functional groups and examine their activity toward ORHP. We find that the quinone groups exhibit very high activity with negligible overpotential.

## Results

### Synthesis and structure characterization

We adopted a two-step method to prepare edge-oxygenated graphitic nanoplatelets. The free edge sites were first created by simultaneously crushing and exfoliating graphite mechanochemically. Then, the reactive edge sites were saturated with mild oxidants of CO_2_ or diluted O_2_. We found that when CO_2_ was applied as an oxidant, carbonyl-related groups were easily formed. In this case, the as-prepared product is designated GNP_C=O_. Even dilute O_2_ could also be selected as an oxidant. Because the electronic ground state of O_2_ and carbon are different, the triplet O_2_ cannot react with singlet defect-free graphitic carbon spontaneously, due to the energy barrier present. This allows selective oxidation of the reactive edge sites with a high degree of controllability. Etheric ring (C–O–C)-functionalized graphitic nanoplatelets (GNP_C–O–C_) were obtained in dilute O_2_ atmosphere.

We first characterized the morphology of the above samples using field emission scanning electron microscopy (FESEM, Supplementary Fig. 1) and transmission electron microscopy (TEM, Supplementary Fig. 2). This analysis showed that GNP_C=O_ and GNP_C–O–C_ had morphologies typical of graphitic nanoplatelets and nanoparticles, respectively. The successful nanosizing and functionalization were further verified by Raman spectra and X-ray powder diffraction (XRD) patterns. As shown in Supplementary Fig. 3, the strong D band (usually as an indicator of defective edges) and broad (002) facets (derived according to the Scherrer equation), together indicate the existence of plenty of edge sites in the nanosized GNPs. The Brunauer–Emmett–Teller (BET, Supplementary Fig. 4) analysis determined that the specific surface areas of GNP_C=O,1_, GNP_C=O,2_, and GNP_C–O–C_, are 450, 753, and 657 m^2^ g^−1^, respectively.

Next, we measured sample oxygen content using three different methods, elemental analysis (EA), energy dispersive spectroscopy (EDS), and XPS. The results (summarized in Supplementary Table 1 and Figs. 5–7) agreed with each other well. Here, we highlight the EA results because it has higher accuracy than the other two methods. The amount of oxygen content was calculated to be 10.5 at% for GNP_C=O,1_, 20.6 at% for GNP_C=O,2_, and 10.0 at% for GNP_C–O–C_. A possible iron remnant was checked by time-of-flight secondary ion mass spectrometry (TOF-SIMS), which has a detection limit as low as ppm. The results (Supplementary Fig. 6) showed that there was no detectable iron in the samples.

Finally, to carefully unravel the nature of the oxygen functional groups we used a combination of techniques, including soft XANES, XPS, FTIR, and CV. Among them, soft XANES is one of the most powerful tools for characterizing graphitic materials, and can provide important information about bonding configurations with high resolution^32–34^.

**Fig. 1:**
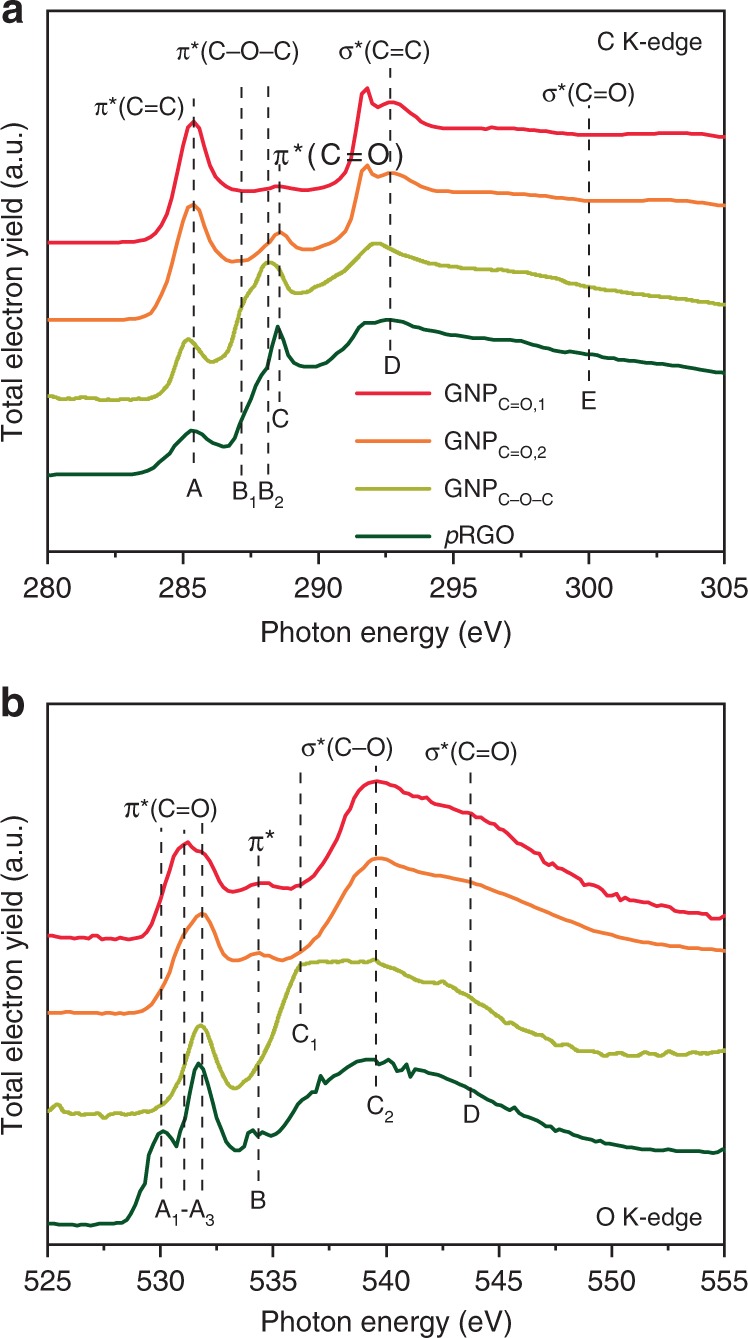
The soft X-ray absorption near-edge structure (XANES). **a** C K-edge. A, 1s – π* from *sp*^2^ C=C; B, 1s – π* from the etheric ring, B_1_, the out-of-plane C–O-C, B_2_, the in-plane C–O-C; C, 1s – π* from ketone or carboxylic acid; D, 1s –  σ* from *sp*^2^ C=C; D, 1s – σ* from C=O. **b** O K-edge. A, 1s – π* from C=O, A_1_, organic carbonate, A_2_, quinone, A_3_, ketone or carboxylic acid; B, the 1s – π* excitations due to the charge transfer between C and O, including C=O and C–O; C, 1s – σ* from C–O, C_1_, etheric ring (C–O–C), C_2_, the C–O in COOH; C, 1s – σ* from C=O. The soft XANES were collected in the total electron yield (TEY) mode.

As shown in Fig. 1a, the C K-edge includes unoccupied π* (peak A–C) and excited σ* (peak D, E) states. Here, we only discuss the fingerprint region of π* in detail. The peak A around 285.4 eV is assigned to 1s – π* from *sp*^2^ C=C^32–36^. The peak B results from the charge transfer induced by the O in the etheric ring. The GNP_C–O–C_ and partially reduced graphene oxide (*p*RGO, Supplementary Methods) exhibit a minor shoulder at 287.2 eV (Peak B_1_), which corresponds to the out-of-plane etheric ring (C–O–C)^32,34,35^. Since the in-plane C–O bonding of C–O–C is much stronger than the out-of-plane, the peak at 288.2 eV is supposed to be in-plane C–O–C at the edge of the GNP. Thus, the GNP_C–O–C_ is mainly composed of in-plane C–O–C, and partly by out-of-plane C–O–C. In *p*RGO there is only out-of-plane C–O–C. The peak C around 288.6 eV is attributed to π*(C=O)^32,35^, which should be determined in combination with other characterizations.

**Fig. 2:**
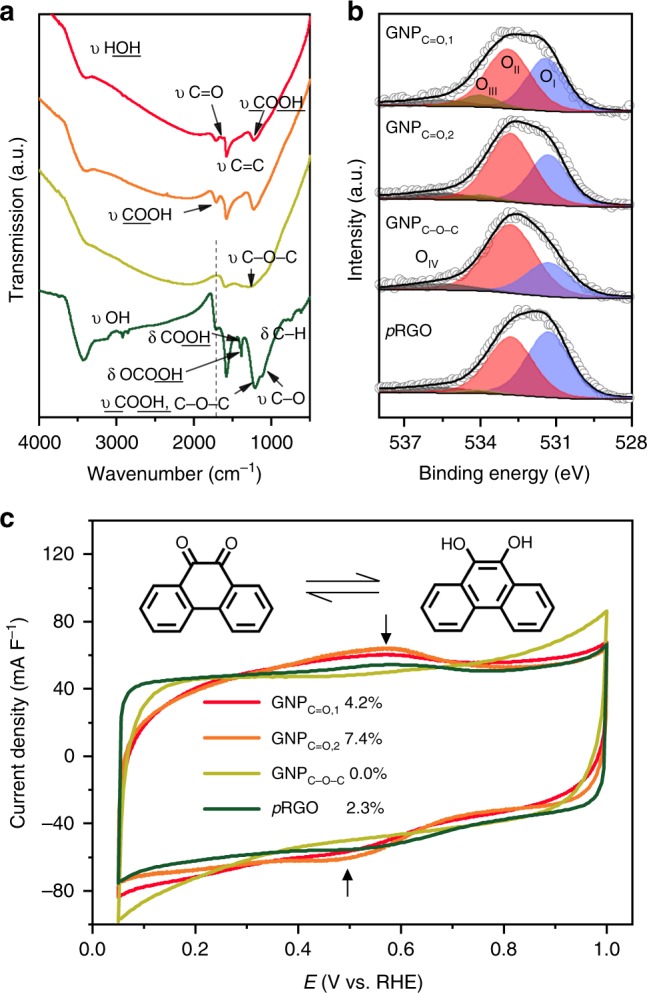
Edge group characterization. **a** Fourier transform infrared (FTIR) spectra. **b** The high-resolution O 1s of the X-ray photoelectron spectra (XPS). O_I_, 531.35 ± 0.05 eV, C=O, quinone, or ketone; O_II_, 532.8 eV, C–O-C, or COOH; O_III_, 534.0 eV, C–O(H); O_IV_, 535.6 eV, adsorbed H_2_O and O_2_. **c** The cyclic voltammetry. The CV curves were measured in Ar-saturated 0.5 M H_2_SO_4_ at a scan rate of 50 mV s^−1^. The quinone redox reaction were recorded in the potential window from 0.3 to 0.8 V.

The soft O K-edge XANES (Fig. 2b) provides further information about the groups. Here, the *p*RGO shows an exclusive A_1_ peak (530.1 eV), which is assigned to the π*(C=O) of the organic carbonate. The peak A_2_ (531.0 eV) is assigned to quinone contributions^33^. The results of the O K-edge demonstrate that the GNP_C=O,1_ is enriched with quinone. The presence of a peak A_2_ shoulder indicates the GNP _C=O,2_ and *p*RGO also contain less quinone groups. Peak A_3_ (531.8 eV) may be composed of ketones or/and carboxylic acid^32–34^. Fortunately, peak A_3_ can be identified by peak C_2_ (539.6 eV), which originates from the σ*(C–O) in carboxylic acid (COOH)^32–34,36^. Peaks A_3_ and C_2_ are dominant in GNP_C=O,2_ and *p*RGO, which indicates that they are mainly composed of COOH.

We can draw the conclusion that the quinone was easily formed in the short reaction time (GNP_C=O,1_). However, carboxylic acid was the main group after the prolonged reaction time (GNP_C=O,2_). The contribution of π* peak B is complicated. It originates from the resonance transition of quinone, or the charge transfer due to the influence of the neighboring O in the etheric ring^33^. The GNP_C–O–C_ gives rise to a σ* resonance at 536.3 eV (peak C_1_), which is attributed to C–O–C^34^. Here, we noticed that GNP_C–O–C_ contains peak A_3_, and a small shoulder of π*(C=O) in the C K-edge, which indicates that some carbonyl-related groups are present in the GNP_C–O–C_.

FTIR is another powerful tool for characterizing functional groups, and was employed here. As shown in Fig. 2a, all of the spectra exhibit a broad band at around 3400 cm^−1^, which is mainly assigned to the stretching vibration (υ OH) of the adsorbed moisture in KBr. Another common band, located at 1610 cm^−1^, is attributed to the asymmetric stretching of *sp*^2^-hybridized C–C bonds (υ C=C). Its shifting and intensity are intimately related to the oxygenated groups^37,38^.

The fingerprint region, ranging from 1750 to 1000 cm^−1^, was scrutinized to identify the functional groups present. The well-documented *p*RGO was analyzed first. Consistent with other reports^38–42^, the functional groups in the *p*RGO were assigned to ketones (υ C=O, the shoulder at 1654 cm^−1^), carboxylic acid (υ C=O at 1715 cm^−1^, δ OH at 1402 cm^−1^, υ C–OH at 1214 cm^−1^), organic carbonate (υ C=O at 1715 cm^−1^, δ OH at 1384 cm^−1^), ethers (υ C–O at 1084 cm^−1^), and aromatic hydrocarbons (δ C–H at 740 cm^−1^)^38–42^.

Both GNP_C=O,1_ and GNP_C=O,2_ produced a typical fingerprint band of carboxylic acid (υ C=O in COOH, 1716 cm^−1^). The COOH is formed via hydrolysis in acid^43,44^. GNP_C=O,1_ and GNP_C=O,2_ also displayed a peak and a shoulder, respectively, at 1632 cm^−1^, which was thought to be the quinone based on the XANES results. The GNP_C–O–C_ displayed a broad band from 1380 to1050 cm^−1^, which was attributed to the asymmetric C–O-C stretching vibration (in-plane υ C–O–C) of the etheric rings. This is caused by an unusual absorption mechanism^40^.

The XPS was then performed (Supplementary Fig. 7). It is known that that the C 1s typically exhibits a pronounced asymmetric tail at higher binding energy^45^, making it difficult to obtain accurate deconvolution results, especially considering the controversial assignment. Here, we only focused on the O 1s spectroscopy.

The high-resolution O 1s are shown in Fig. 2b. After careful deconvolution with the same standard rules, four distinct regions were identified. To easily distinguish them, we labelled them O_I_ (531.35 ± 0.05 eV, C=O related groups)^46,47^, O_II_ (532.8 eV, C–O–C or COOH)^46,47^, O_III_ (534.0 eV, C–O(H))^46^, and O_IV_ (535.6 eV, physically adsorbed H_2_O and O_2_)^41,42^. Because the different groups overlap, it was difficult to determine the contents of the groups by O 1s alone. However, we can still draw some useful information. All samples had a very small O_III_ region, which indicates that very little phenolic -OH is present. Based on the XANES and FTIR results, it was determined that the O_II_ regions in GNP_C=O,1_, GNP_C=O,2_, and *p*RGO mainly result from COOH. However, the O_II_ region in GNP_C-O-C_ originates from C–O-C. GNP_C=O,1_ had a higher percentage of O_I_ relative to GNP_C=O,2_ due to the higher content of quinone. The GNP_C-O-C_ exhibited the smallest O_I_ region. In contrast, the *p*RGO exhibited the highest O_I_ region. However, the ketone and quinone in the O_I_ region still could not be distinguished.

Fortunately, quinone is sensitive to cyclic voltammetry (CV). Since the quinone redox couple is the only reaction that can be detected by CV, we used CV to characterize the content of quinone in the samples (Supplementary Fig. 8)^10,31^. As shown in Fig. 2c, the quinone content was 4.2%, 7.4%, 0.0%, and 2.3% for GNP_C=O,1_, GNP_C=O,2_, GNP_C–O–C_, and *p*RGO, respectively. The absence of quinone redox indicates that the O_I_ region in GNP_C–O–C_ is attributed to the presence of ketone. The reduced quinone redox content in *p*RGO demonstrates that most of the O_I_ region is composed of COOH. The functional groups of the samples are summarized in Supplementary Table 2.

### Oxygen reduction to hydrogen peroxide

The oxygen reduction to hydrogen peroxide (ORHP) performance of the synthesized samples with different oxygen groups was evaluated using a rotating ring-disk electrode (RRDE). Since H_2_O_2_ can upshift the potential of the reference electrode because of its strong oxidizing nature (Supplementary Fig. 9), a salt bridge must be used to separate the electrochemical cell and reference electrode (Supplementary Fig. 10). For a fair comparison of activity, all of the tested samples were tuned to the same capacitance by changing the volume of drop-cast ink. We used a reversible hydrogen electrode (RHE, Supplementary Fig. 11) for all our analyses.

The polarization curves were measured in O_2_-saturated 0.1 M aq. KOH solution at a scan rate of 1600 rpm. To eliminate the contributions of capacitance, we averaged the current of the forward and backward scans. The H_2_O_2_ current (*J*_H2O2_) was collected at the ring electrode (*J*_R_) with an applied potential of 1.15 V, and the collection efficiency was 37% (Supplementary Fig. 12). The four-electron byproducts of H_2_O were calculated using the relationship: *J*_H2O_ = *J*_D_ – *J*_R_.

**Fig. 3:**
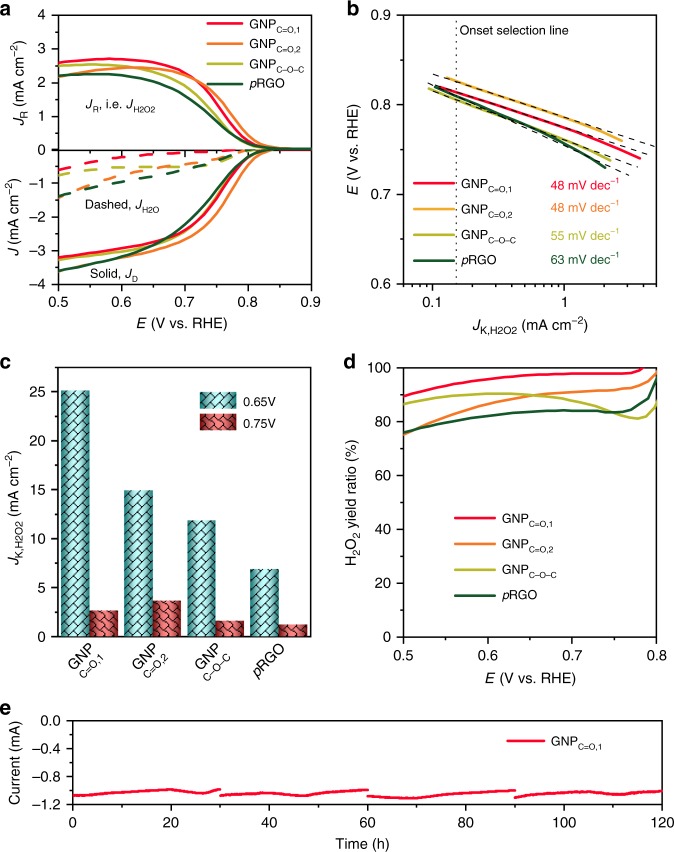
The performance characterizations of ORHP. **a** The polarization curves of H_2_O_2_ current (*J*_R_ or *J*_H2O2_), disk current (*J*_D_), and H_2_O current (*J*_H2O_). The curves were measured in O_2_-saturated 0.1 M KOH solution at a scan rate of 10 mV s^−1^ by RRDE with a rotation speed of 1600 rpm. The applied potential of the ring was 1.15 V. The current was the average of the forward and backward scans. **b** The corresponding Tafel plot of kinetic current of H_2_O_2_ (*J*_K,H2O2_). **c** The *J*_K,H2O2_ comparison at 0.65 and 0.75 V. **d** The corresponding H_2_O_2_ yield ratio. **e** Stability tests in four fresh 0.1 M KOH electrolytes, which were measured in a H-type cell, with 400 ppm MgSO_4_ as stabilizer to suppress the decomposition of H_2_O_2_. The applied potential was 0.65 V.

As shown in Fig. 3a and Supplementary Table 3, the carbonyl-enriched samples, GNP_C=O,1_ (0.826 V), GNP_C=O,2_ (0.815 V), and *p*RGO (0.810 V), exhibited a higher onset potential than GNP_C–O–C_ (0.805 V). Here, the onset potential is defined as the potential measured at a current density of 0.15 mA cm^−2^ (5% of the theoretical limiting current) for ORHP. The result indicates that the sample with the in-plane etheric ring had lower activity than the carbonyl-enriched samples.

It is noteworthy that all of the onset potentials were even higher than the thermodynamic equilibrium potential (O_2_ + H_2_O + 2 *e* ⇌ HO_2_^−^ + OH^−^, 0.75 V vs RHE). This counterintuitive phenomenon has been widely reported^16,48^. Bao and his colleagues attributed this to a Nernst-related potential shift due to the low concentration of H_2_O_2_ in the electrolyte, and/or a possible pH-related change^48^. Although H_2_O_2_ can upshift the potential of the reference electrode, its influence can be easily ruled out by employing a salt bridge and refreshing the electrolyte for every measurement. This abnormal phenomenon did not exist with a planar electrode, which further confirms that the potential shifting is unrelated to the yield of H_2_O_2_. We suggest this abnormal phenomenon only results from the localized pH-related change in the mesoporous electrode due to the constraint of mass transport.

The kinetic current of H_2_O_2_ (*J*_K,H2O2_) was calculated according to the Koutecky-Levich equation: 1∕*J*_H2O2_ = 1∕*J*_K,H2O2_ + 1∕*J*_L,H2O2_, where *J*_K,H2O2_ is the measured current of the H_2_O_2_ yield, *J*_L,H2O2_ is the theoretical limiting current of ORHP^23,49^. *J*_L,H2O2_ was obtained from the Levich equation and determined to be 2.9 mA cm^−2^. The calculated results are shown in the Tafel plot in Fig. 3b. GNP_C=O,1_, and GNP_C=O,2_ showed the smallest Tafel slope (48 mV dec^−1^), which was superior to the C–O–C-enriched GNP_C–O–C_ (55 mV dec^−1^). The same Tafel slope indicates similar active sites. Although the *p*RGO was also carbonyl-enriched, its Tafel slope was only 63 mV dec^−1^. This is possibly due to the considerable N content, which is inevitably introduced during the preparation of *p*RGO. The doped N is known to enhance the yield of 4e product H_2_O^50^.

The *J*_K,H2O2_ at 0.65 and 0.75 V were selected to compare activity (Fig. 3c). We note that GNP_C=O,1_ had the highest activity (25.1 mA cm^−2^) at 0.65 V. However, the most active sample was GNP_C=O,2_ (3.6 mA cm^−2^) at a higher potential of 0.75 V. This phenomenon is caused by the samples′ different selectivity (the H_2_O_2_ yield ratio, Fig. 3d), which plays a major role in the low potential region. The GNP_C=O,1_ exhibited higher selectivity (the H_2_O_2_ yield ratio, 97.8% at 0.75 V) with an electron transfer number of nearly 2 (Supplementary Fig. 13), which is the highest ratio reported so far (Supplementary Table 3)^16,18,19,21,22,25–29,48^. The *p*RGO exhibited the poorest *J*_K,H2O2_ (1.2 mA cm^−2^ at 0.75 V) and the lowest H_2_O_2_ yield ratio (83.4% at 0.75 V) because of the presence of graphitic N (Supplementary Fig. 14), which is reported to be the active sites for 4*e* ORR^50^.

The H_2_O_2_ yield ratio of COOH-enriched GNP_C=O,2_ decreased relative to the quinone-enriched GNP_C=O,1_. The possible reason is that COOH has a negative impact on selectivity. The H_2_O_2_ yield ratio of all of the carbonyl-enriched samples decreased as we lowered the potential. This may have occurred due to the reduction of quinone to hydroquinone at low potential. Distinctively, the GNP_C–O–C_ displayed a tendency opposite to the others, due to the absence of quinone.

The ORHP performance was also evaluated in neutral (0.05 M Na_2_SO_4_) and acidic (0.1 M HClO_4_) media. The results are shown in Supplementary Fig. 15. Both results in neutral and acidic media demonstrated that the quinone-enriched sample (GNP_C=O,1_) had the highest selectivities, which were 95% (0.6 V in neutral) and 85% (0.2 V in acid), respectively. In the neutral medium, the onset potential is the same with the thermodynamic equilibrium potential (0.70 V), which indicates the absence of potential shift, which is caused by the localized *p*H-related change. In the acidic medium, the activity toward ORHP is poor. The result agrees well with the previous reports^16–18^.

Besides selectivity and activity, stability is also one of the three indispensable factors for catalysts, and it affects their economical durability. The samples′ stability was measured in an H-type cell, which was separated by a Nafion 115 membrane (Supplementary Fig. 16). It is known that the alkaline H_2_O_2_ solution is unstable, and spontaneous decomposition will occur^1,51,52^. Approximately 30% of H_2_O_2_ was decomposed during a 30 h stability test (Supplementary Fig. 17). Therefore, MgSO_4_, a popular stabilizer in alkaline solution, was selected to suppress the decomposition^51^.

As shown in Fig. 3e, the current exhibited a slow decline as the time increased. The possible reason is that the increasing H_2_O_2_ concentration enhances electrolyte viscosity. The increasingly sluggish diffusion of O_2_ in the H_2_O_2_ solution subsequently deteriorates the ORHP current^53^. This speculation was further verified by the observation that there was no current drop after changing to a fresh electrolyte. Even after 120 h, there was no evident decline, which demonstrates that the catalyst has good stability.

The concentration of generated H_2_O_2_ was determined using the KMnO_4_ titration method (Supplementary Movie 1)^28^. With an applied potential of 0.65 V in 90 mL electrolyte, a typical concentration of H_2_O_2_ after 30 h reaction is about 6.1 mM, with a Faraday efficiency^11,23^ of 95%.

### Determination of active sites

So far, we have shown that the carbonyl-enriched groups possess higher activity than the etheric ring groups. However, the nature of the active carbonyl groups remains elusive. To elaborate the nature of the active carbonyl groups, we designed a special experiment.

**Fig. 4:**
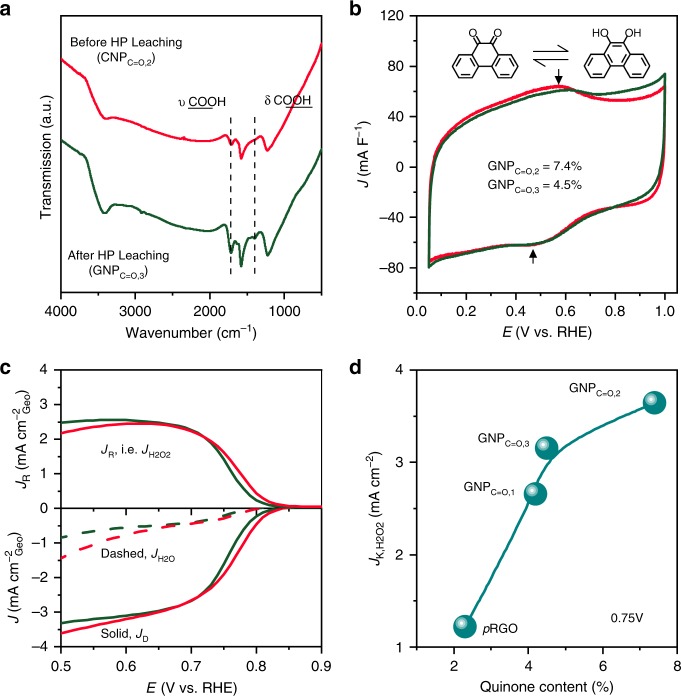
Determination of active sites. **a** FTIR. The COOH is increased. **b** Cyclic voltammetry, which was measured in Ar-saturated 0.5 M H_2_SO_4_ at a scan rate of 50 mV s^−1^. The quinone is decreased. **c** The polarization curves of *J*_H2O2_, *J*_D_, and *J*_H2O_. The curves were measured in O_2_-saturated 0.1 M KOH solution at a scan rate of 10 mV s^−1^ by RRDE with a rotation speed of 1600 rpm. The current was the average of the forward and backward scan. **d** The *J*_K,H2O2_ as a function of quinone content.

We tuned the content of quinone and carboxylic acid by leaching the sample of GNP_C=O,2_ in an acidified concentrated H_2_O_2_ solution. The leached sample was labelled GNP_C=O,3_. As shown in Fig. 4a, FTIR determined that the carboxylic acid content evidently increased after leaching. However, the quinone content was reduced from 7.4% to 4.5% (Fig. 4b). This change in groups was also recorded by XPS (Supplementary Fig. 18). The active sites can be easily identified by comparing the ORHP performance of GNP_C=O,2_ and GNP_C=O,3_.

The polarization curves are shown in Fig. 4c. The GNP_C=O,3_ had both a lower onset potential of 14 mV and *J*_K,H2O2_ (3.1 mA cm^−2^, Supplementary Fig. 19) than GNP_C=O,2_. The relation of *J*_K,H2O2_ and quinone content was further checked, and the results are shown in Fig. 4d. *J*_K,H2O2_ increased as the quinone content increased. Because of the influence of other groups, it did not follow a linear relation. These results confirmed that the active sites were quinone.

**Fig. 5:**
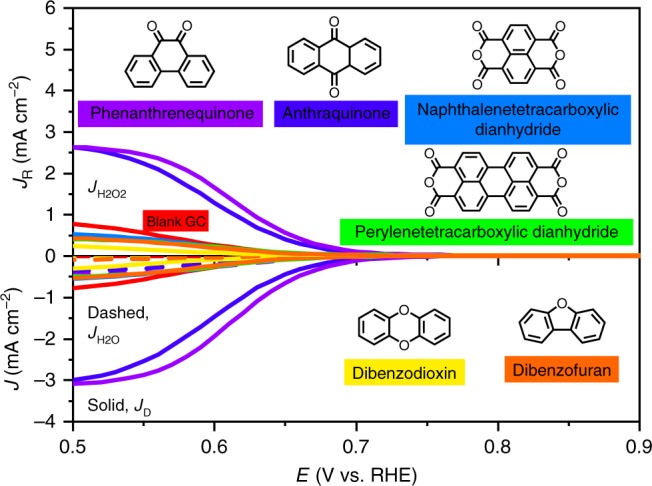
The ORHP performance of standalone molecules. Phenanthrenequinone, anthraquinone, naphthalenetetracarboxylic dianhydride, perylenetetracarboxylic dianhydride, dibenzodioxin, dibenzofuran, and blank glass carbon (GC) were compared. The curves were measured in O_2_-saturated 0.1 M KOH solution at a scan rate of 10 mV s^−1^ by RRDE with a rotation speed of 1600 rpm. The applied potential of the ring was 1.15 V. The current was the average of the forward and backward scans.

To further verify these results, we investigated several standalone molecules with quinone, carboxylic acid, and etheric ring groups, such as phenanthrenequinone, anthraquinone, naphthalenetetracarboxylic dianhydride, perylenetetracarboxylic dianhydride, dibenzodioxin, and dibenzofuran. The polarization curves are shown in Fig. 5. Except for phenanthrenequinone and anthraquinone, the other four molecules did not show activity towards ORHP; the activity was inferior to blank glass carbon (GC). The phenanthrenequinone was superior to anthraquinone in both the *J*_K,H2O2_ (0.7 vs 0.5 mA cm^−2^ at 0.65 V) and Tafel slope results (45 vs 48 mV dec^−1^, Supplementary Fig. 20). These molecular chemistry results further confirm that the quinones are the active sites.

### Theoretical investigation

**Fig. 6:**
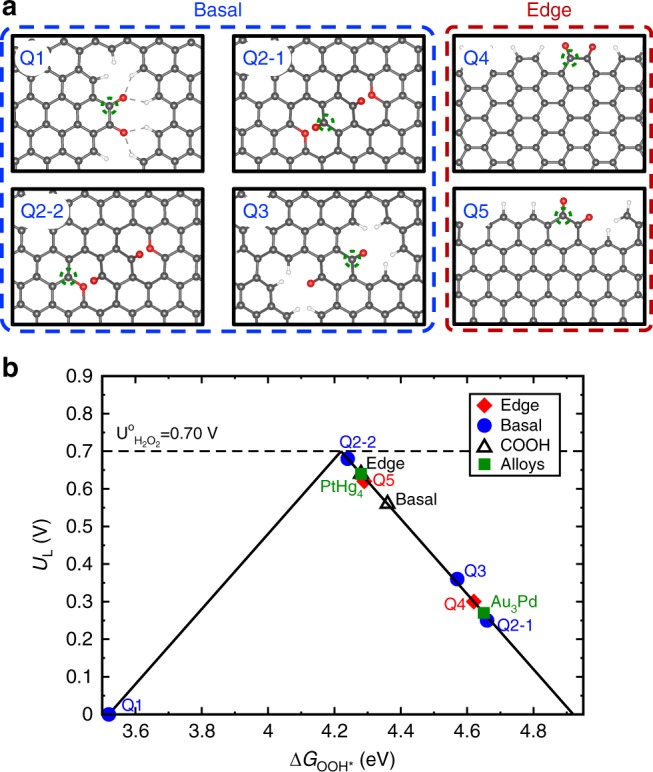
Theoretical analysis of different oxygenated groups. **a** The atomic structures of the examined oxygen functional groups. Color code: carbon, gray; oxygen, red; hydrogen, white. The corresponding examined active sites are marked with a dashed green circle in each model structure. **b** Theoretical ORHP activity volcano plot. Horizontal dashed line corresponds to the thermodynamic equilibrium potential for ORHP (*U*^0^ = 0.70 V). The activity of alloys and edge COOH are adapted from refs. ^1,16^, respectively.

To gain atomistic insights about the nature of the active quinone motifs, we next used density functional theory (DFT) calculations. We examined a variety of model structures (Fig. 6a) to study the different possible quinone groups on the edge and basal planes. These model structures were used to model the ORHP reaction pathway (Eqs. 1 and 2)^5,16^:

1 $${\mathrm{O}}_2 + {\mathrm{H}}_2{\mathrm{O}} + {\mathrm{e}}^-- + \ast \to {\mathrm{OOH}} \ast + {\mathrm{OH}}^--$$

2 $${\mathrm{OOH}} \ast + e^-- \to {\mathrm{HO}}_2^ - + \ast $$

where the O_2_ molecule adsorbs at the carbon surface and is reduced through the first proton-electron transfer to form OOH* (Eq. 1). The second electron transfer results in the formation of HO_2_^–^, which is desorbed from the surface (Eq. 2). The key intermediate OOH* plays a pivotal role in the ORHP. Its adsorption in Eq. 1 and desorption in Eq. 2 jointly determines the activity, according to the Sabatier principle^1,16^. The adsorption energy of OOH* (Δ*G*_OOH*_) is therefore the best descriptor to capture the trends in activity for different oxygen functional groups^1,16^.

We also calculated the barrier of proton transfer to the oxygen atom of adsorbed OOH* to be zero. This means that there is a close connection between the thermodynamic and kinetic formulations for two-electron oxygen reduction reaction. Therefore, we only focus on thermodynamic analysis, which has played an essential role in providing insights on the nature of active sites and guiding the design and optimization of various catalysts.

In line with previous reports^1,16^, we used the calculated limiting potential (*U*_L_) as the indicator of activity towards ORHP, which is defined as the maximum potential at which the above two reaction steps are downhill in free energy. The ORHP results in this work on quinone functional groups are summarized along with the previous report of oxygen functional groups by Lu, *et al*.^16^ in an activity volcano plot in Fig. 6b. The vertex of the activity volcano corresponds to the thermodynamic equilibrium potential (*U*^0^ = 0.70 V) for the ORHP. In theory, an ideal catalyst should have a Δ*G*_OOH*_ of 4.22 (±0.1) eV, which provides the highest activity. Based on this analysis, while the quinone functional groups on the edge (Q-edge 5) are comparable to the reported catalysts for ORHP^1,16^, the Q-basal 2-2 displayed the highest activity. Of note, the formation of quinone functional groups on the edge seems more feasible than in the basal area, because the formation of Q-Basal groups significantly interrupts the *sp*^2^ network and requires a lot of energy input. However, the edge-located structures of Q-Edge 4 and 5 are easily formed, because the *sp*^2^ C–C bond breaking is lower. Thus, the Q-Edges are the most likely sites.

Furthermore, a different mechanism similar to industrial anthraquinone process was considered. The results of the anthraquinone mechanism shown in Supplementary Figs. 21 and 22 reveals that the formation of anthrahydroquinone (AHQ) is uphill by 3.64 eV. In addition, the next step, which is transferring proton from of AHQ to O_2_ molecule and forming radical OOH, is even more exergonic around 4.93 eV. Both of them are far beyond the energy capacity for the two-electron ORR. Therefore, the anthraquinone mechanism is not a possible competitive pathway for our catalyst system.

## Discussion

In summary, we adopted a pre-activated method to decorate the dangled edges of graphitic materials with targeted groups (ether, carboxyl and quinone). The functional groups were then characterized by a combination of soft XANES, XPS, FTIR and CV. Our results confirmed a new class of quinone-edged groups, which exhibited higher selectivity than previously reported oxygenated groups with similar onset potential. The quinone-enriched samples (GNP_C=O,1_) exhibited a H_2_O_2_ yield ratio of 97.8% at 0.75 V. The results were further verified using standalone molecular chemistry and theoretical analysis. These findings will be beneficial for understanding active sites in ORHP, and will be a guide to designing high ORHP catalysts.

## Methods

### Preparation of graphitic nanoplatelets

The pre-activated method was used for the preparation of the graphitic nanoplatelets (GNP). We first cleaved graphite using the mechanochemical method. The graphite crushing and exfoliation into nanosized particles were accomplished at the same time. The freshly broken edges are free and reactive. Then, the activated edges were reacted with target molecules, such as CO_2_ and O_2_. The groups were edge-enriched with CO_2_ and O_2_, and the resulting as-prepared GNPs were designated as GNP_C=O_ and GNP_C–O–C_, respectively.

GNP_C=O,1_: the mechanochemical ball-milling method was employed to activate and saturate the graphite at the same time. The preparation was conducted on a planetary ball-milling device (Pulverisette 6, Fritsch GmbH) in a rotation speed of 500 rotation per minutes (rpm). In brief, graphite (15 g, Alfa Aesar, 100 mesh, 99.9995%, product number: 14735), dry ice (100 g, Hanyu Chemical Inc.), and hardened steel balls with a diameter of 3 mm (500 g) were placed into a ball-mill container (250 mL). Then, the air in the container was completely pumped out by five repeated argon (Ar) charging/discharging cycles for 15 min and ball-milled under reduced pressure for 15 h. Finally, the as-prepared GNP_C=O_ was leached in 1 M aq. H_2_SO_4_ solution for 24 h to completely remove possible contamination from unbound Fe debris, followed by rinsing with ultra-pure water (18.2 MΩ cm, Direct-Q^®^ 3UV, Millipore Corporation) more than 6 times and freeze-drying in *tert*-butyl alcohol. Finally, the samples were further dried in vacuum oven at 80 °C for 10 h.

GNP_C=O,2_: the amount of carbonyl-related group loading was controlled by varying the graphite loading amounts and ball-milling conditions. GNP_C=O,2_ was prepared with more carbonyl-related groups by ball-milling graphite (10 g) and dry ice (100 g) with hardened steel balls (500 g, *Φ* = 5 mm) for 40 h.

GNP_C=O,3_: the different contents of carbonyl-related groups was obtained by H_2_O_2_ leaching in acid. The leaching process was conducted by immersing GNP_C=O,2_ in 20 mL 3.5 M aq. H_2_O_2_ and 1.0 M acetic acid mixture for 12 h. Here, acetic acid acted as a stabilizer to suppress the spontaneous decomposition of H_2_O_2_. Finally, the samples were further dried in a vacuum oven at 80 °C for 10 h before characterizations.

GNP_C-O-C_: the mechanochemical ball-milling method was first applied for activation. The experiment procedures were conducted in a planetary ball-milling device (Pulverisette 6, Fritsch GmbH) at a rotation speed of 500 rpm with the protection of 5 bar Ar (UHP, 99.999%, N50, KOSEM, Korea). In brief, graphite (15 g) and hardened steel balls (500 g, *Φ* = 3 mm) were charged in a ball-mill container (250 mL). Then, the container was filled with argon gas (5 bar), after five purging cycles with the aid of a vacuum pump to remove residual air.

After cooling to room temperature, the container was filled with an O_2_/Ar mixture (10 vol%) for 6 h at a flow rate of 250 standard cubic centimeters per minute (sccm). SAFETY NOTE: The concentration of O_2_ in the gas mixture should be lower than the burn-off point to avoid fire, which can be caused by violent oxidation.

Since the dangling edges activated by unzipping the graphitic framework tend to reconstruct spontaneously to reduce their surface energy, the cleavage process was exponentially reduced as the ball-milling was prolonged. Gas oxidation in the O_2_/Ar mixture was divided into 7 periods. The ball-milling time for each period was 20 min, 20 min. 20 min, 30 min, 30 min, 60 min, and 120 min, respectively. The total ball-milling time was 5 h.

Finally, to completely remove unbound Fe debris, the as-prepared GNP_C–O–C_ was leached in 1 M aq. H_2_SO_4_ solution for 24 h, followed by rinsing with ultra-pure water more than six times and freeze-drying in *tert*-butyl alcohol. Finally, the samples were further dried in a vacuum oven at 80 °C for 10 h.

### Structural characterization

The microstructures were characterized on a JEM-2100 transmission electron microscope (TEM) at an accelerating voltage of 200 kV, and by field emission scanning electron microscopy (FESEM, Nova NanoSEM, FEI), equipped with energy dispersive spectroscopy (EDS, EDAX, AMETEK). X-Ray diffraction (XRD) patterns were recorded on a D/max2500V (Rigaku, Japan) using Cu-Kα radiation (40 kV, 100 mA, *λ* = 1.5418 Å) in a 2θ range of 3°–60° at a scan rate of 4° min^−1^. The specific surface area was analyzed on a Micromeritics ASAP 2504 N by nitrogen adsorption-desorption isotherms using the Brunauer-Emmett-Teller (BET) method. The pore distributions were calculated by the non-local density functional theory (NLDFT) method.

Fourier transform infrared spectra (FTIR) were collected on a Perkin-Elmer Spectrum 100 with a resolution of ~1 cm^−1^, and the samples were tableted with KBr as support. The Raman spectra were characterized on a WITec Alpha300R with a laser wavelength of 532 nm.

The elemental analysis (EA) was conducted using a Flash 2000 CHNS/O Analyzers (Thermo Scientific). All samples were measured at least three times. The element of iron was detected by time-of-flight secondary ion mass spectrometry (TOF-SIMS, TOF.SIMS^5^, IONTOF GmbH, Germany) with a resolution of ppm). The primary ion species was Bi with a dose of 2.0 × 10^9^, and the raster area was about 400 × 400 µm^2^. The X-ray photoelectron spectra (XPS) was recorded on a Thermo Fisher XPS spectrometer (K-alpha), which employed monochromatic Al Ka radiation as the X-ray source.

The soft X-ray absorption near edge structure (XANES) experiments were performed at the BL12B-A beamline in the National Synchrotron Radiation Laboratory (NSRL), University of Science and Technology of China (USTC), Hefei, P. R. China.

### Electrochemical measurements

The electrochemical measurements were conducted in a three-electrode electrochemical cell on a workstation of CompactStat (Ivium Technologies B.V., Netherlands). A graphite rod (Alfa Aesar, Ultra purity, 99.9995 %) and an Ag/AgCl electrode were selected as the counter electrode and reference electrode, respectively. As-prepared GNP_C=O,1_, GNP_C=O,2_, GNP_C=O,3_, GNP_C-O-C_, and *p*RGO inks were drop-cast on glassy carbon (GC, 0.247 cm^2^) supports to prepare working electrodes.

To obtain reliable and reproducible measurements, the cleanness of the GC supports is particularly important. Before each measurement, the cleanness of blank GC was first checked by scanning CV at a scan rate of 50 mV s^−1^. The current curves should only exhibit the shape of capacitance with a current density less than an order of magnitude below 10^−7^ A. If it was not, the GC support was polished with alumina (0.05 μm), and then ultrasonically cleaned in ethanol and ultra-pure water.

### Computational method

We used the Atomic Simulation Environment (ASE)^54^ to handle the simulation and the QUANTUM ESPRESSO^55^ program package to perform electronic structure calculations. The electronic wavefunctions were expanded in plane waves up to a cutoff energy of 500 eV, while the electron density is represented on a grid with an energy cutoff of 5000 eV. Core electrons were approximated using ultrasoft pseudopotentials^56^. To describe chemisorption properties on graphene structures, we used the PBE exchange-correlation functional with dispersion correction^57^. Graphene structures were modeled as one layer with a vacuum of 20 Å to decouple the periodic replicas. To model the quinone functional groups in the basal plane, we use a 5 × 5 super cell lateral size, and the Brillouin zone was sampled with (4 × 4 × 1) Monkhorst-Pack k-points. For the oxygen functional groups in the edge, we used a super cell with a lateral size 5 × 6 and the Brillouin zone was sampled with a (1 × 4 × 1) Monkhorst-Pack k-points.

## Supplementary information

Supplementary Information

Peer Review File

Description of Additional Supplementary Files

Supplementary Movie 1

## Data Availability

The data that support the findings of this study are available from the corresponding author upon reasonable request.
