# Supplementary material for: Building and identifying highly active oxygenated groups in carbon materials for oxygen reduction to H2O2
Source: Nat Commun. 2020 May 5;11:2209. doi: 10.1038/s41467-020-15782-z (PMC7200778; doi:10.1038/s41467-020-15782-z)
Supplement: Supplementary file 1 — Supplementary Information [file 41467_2020_15782_MOESM1_ESM.pdf]

Supplementary Information

Building and identifying highly active oxygenated groups in carbon materials for oxygen reduction to H_2O_2

Han *et al.*

Supplementary Information

Building and identifying highly active oxygenated groups in carbon materials for oxygen reduction to H_2O_2

Gao-Feng Han[1], Feng Li[1]*, Wei Zou[2], Mohammadreza Karamad[3], Jong-Pil Jeon[1], Seong-Wook Kim[1], Seok-Jin Kim[1], Yunfei Bu[4], Zhengping Fu[2,5], Yalin Lu[2,5], Samira Siahrostami[6]* and Jong-Beom Baek[1]*

[1] *School of Energy and Chemical Engineering/Center for Dimension-Controllable Organic Frameworks, Ulsan National Institute of Science and Technology (UNIST), Ulsan 44919, South Korea.*

[2] *CAS Key Laboratory of Materials for Energy Conversion, Department of Materials Science and Engineering, University of Science and Technology of China (USTC), Hefei 230026, P. R. China.*

[3] *Department of Chemical and Petroleum Engineering, University of Calgary, 2500 University Drive NW, Calgary, Alberta T2N 1N4, Canada*

[4] *Jiangsu Key Laboratory of Atmospheric Environment Monitoring and Pollution Control, School of Environmental Science and Engineering, Nanjing University of Information Science and Technology (NUIST), 219 Ningliu, Nanjing, Jiangsu 210044, P. R. China*

[5] *Synergetic Innovation Center of Quantum Information and Quantum Physics and Hefei National Laboratory for Physical Sciences at Microscale, University of Science and Technology of China (USTC), Hefei 230026, P. R. China.*

[6] *Department of Chemistry, University of Calgary, 2500 University Drive NW, Calgary, Alberta T2N 1N4, Canada*

* To whom correspondence should be addressed: E-mail: jbbaek@unist.ac.kr, samira.siahrostami@ucalgary.ca, lifeng@unist.ac.kr.

Supplementary Figures:

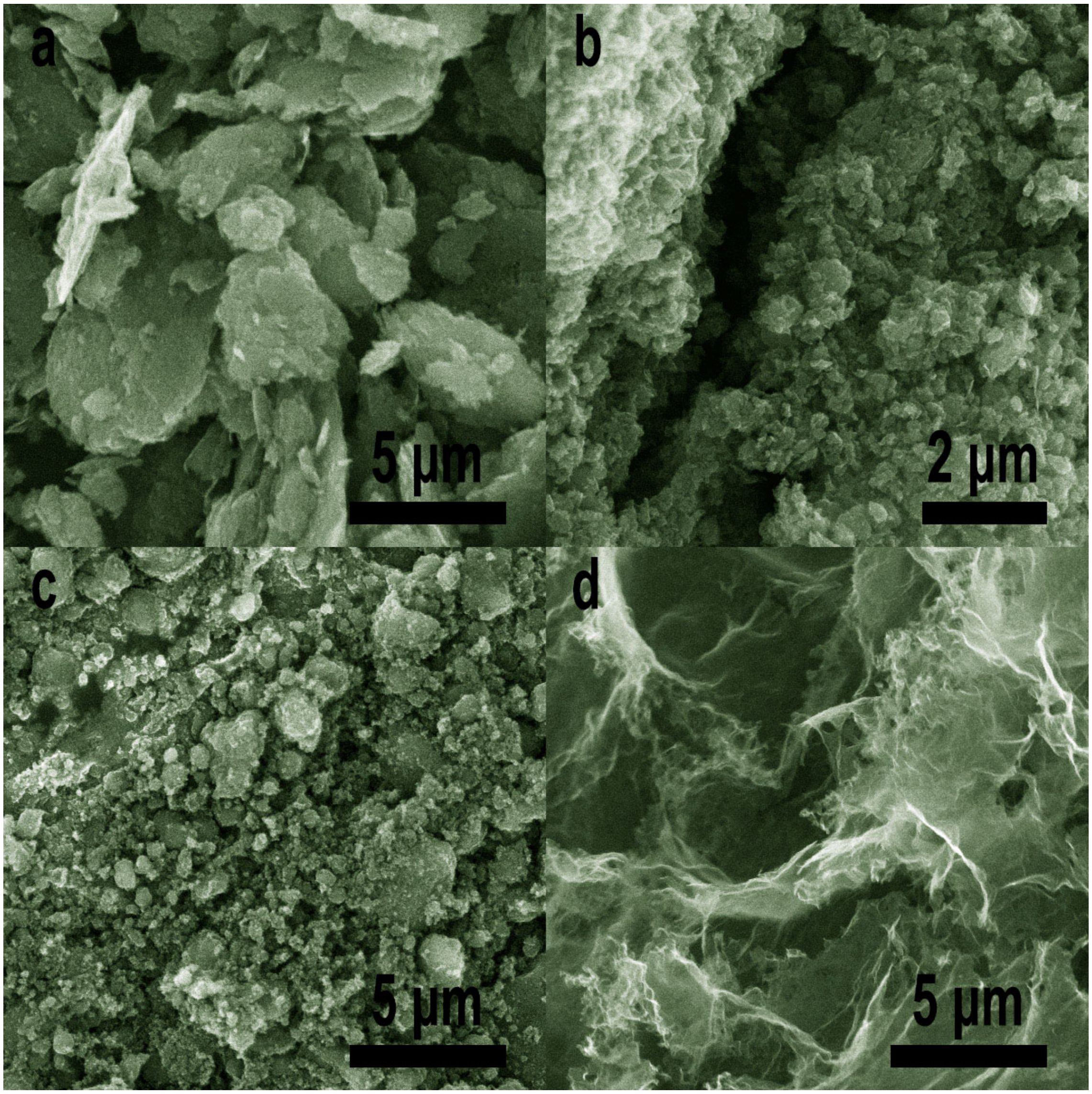

Supplementary Fig. 1 | Field-emission scanning electron microscopy (FE-SEM) images of samples. **a**, $GNP_{C=O,1}$. **b**, $GNP_{C=O,2}$. **c**, $GNP_{C\text{-}O\text{-}C}$. **d**, custom synthesized partially reduced graphene oxide (*p*RGO).

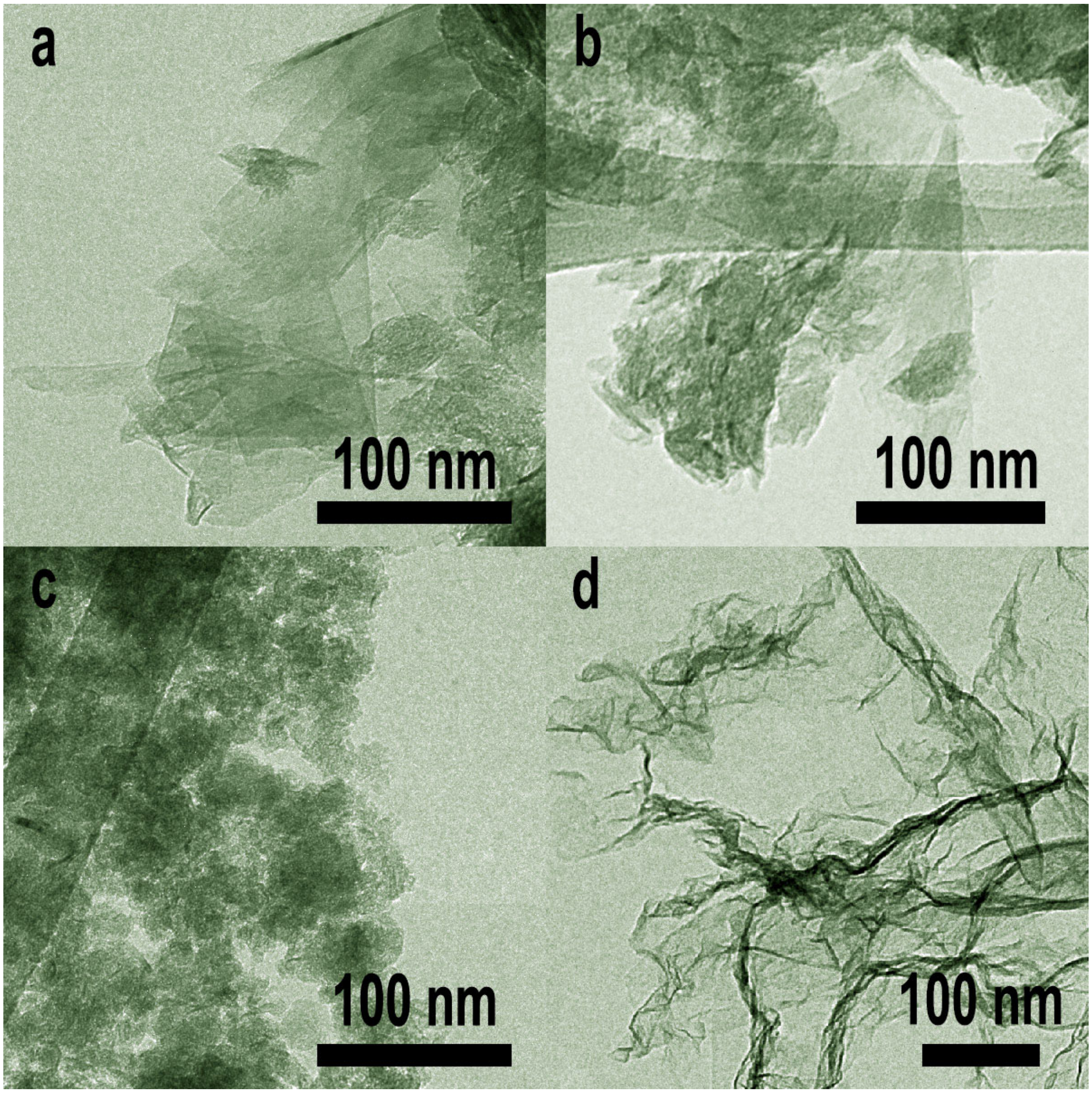

Supplementary Fig. 2 | Transmission electron microscopy (TEM) images of samples. **a**, $GNP_{C=O,1}$. **b**, $GNP_{C=O,2}$. **c**, $GNP_{C\text{-}O\text{-}C}$. **d**, *p*RGO.

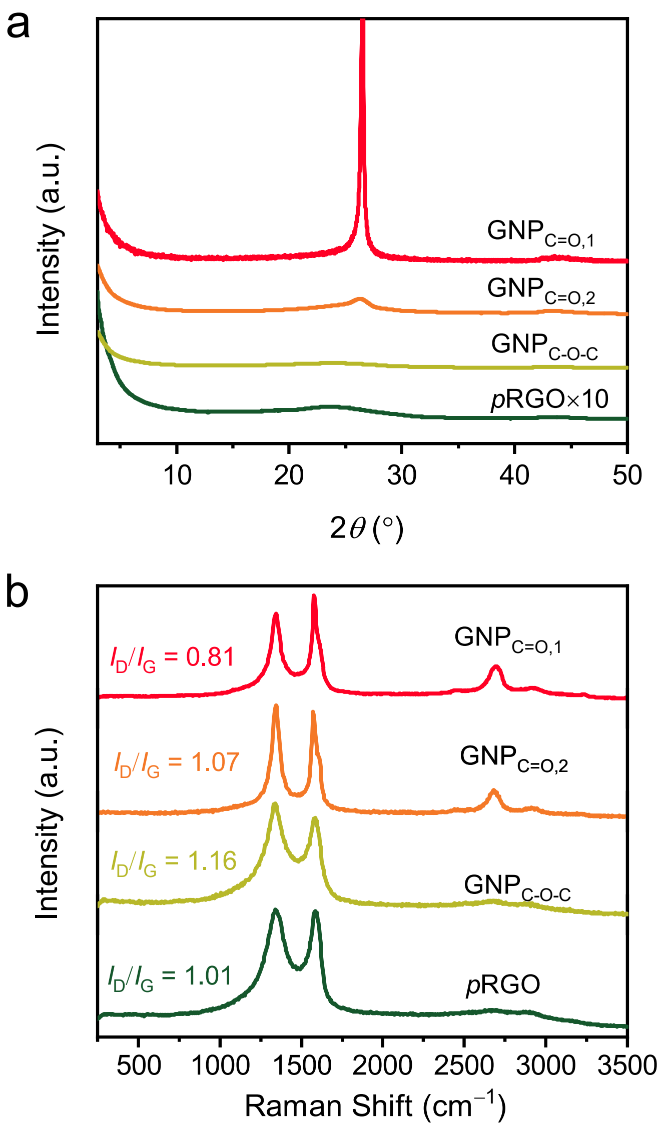

Supplementary Fig. 3 | Structural characterizations of graphitic nanoplatelets ($GNP_{C=O,1}$, $GNP_{C=O,2}$, and $GNP_{C\text{-}O\text{-}C}$) and custom synthesized partially reduced graphene oxide (*p*RGO). **a**, XRD patterns. **b**, Raman spectra.

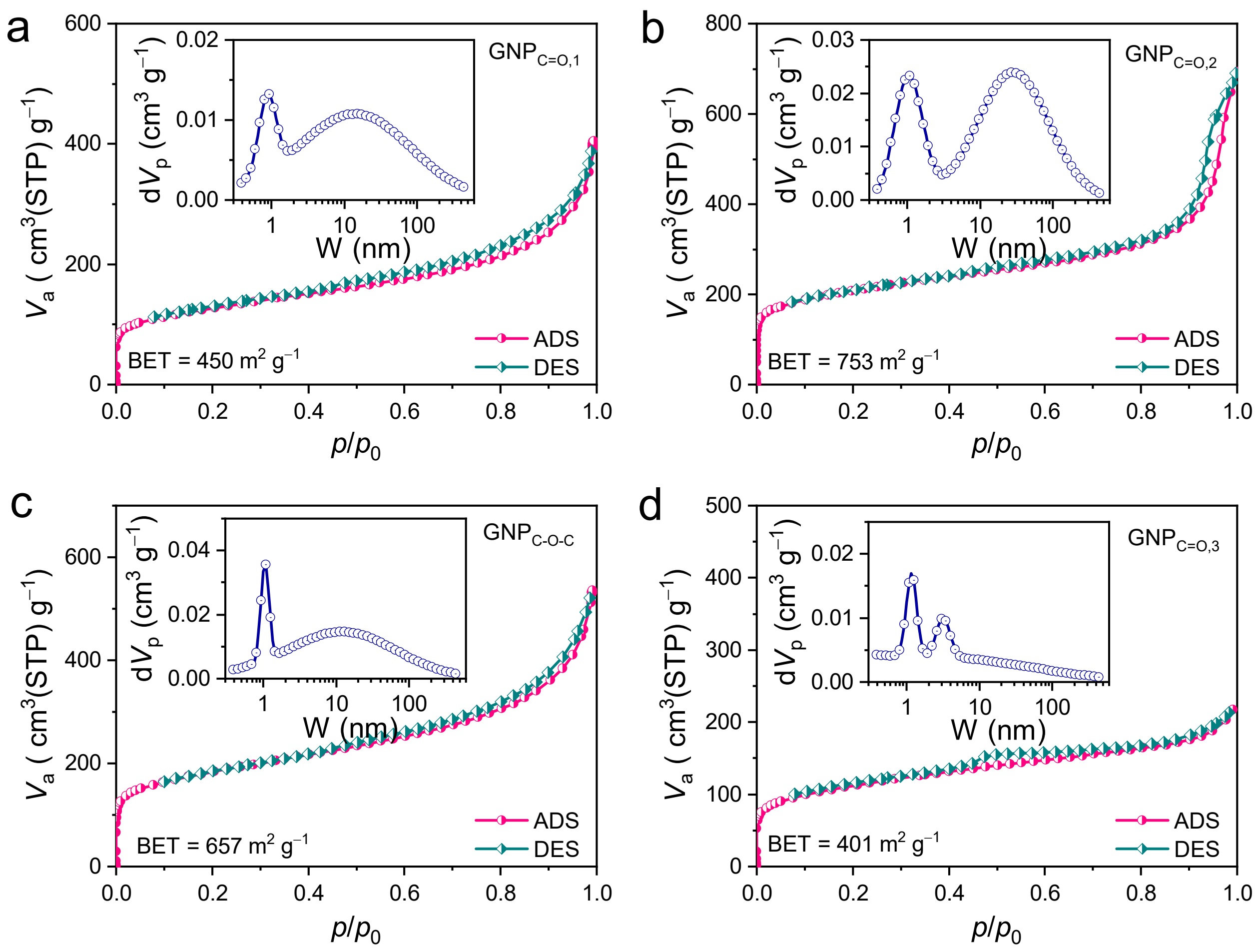

Supplementary Fig. 4 | The Brunauer–Emmett–Teller (BET) measurements. **a**, GNP$_{C=O,1}$, **b**, GNP$_{C=O,2}$, **c**, GNP$_{C-O-C}$, and **d**, GNP$_{C=O,3}$. The insets are the pore size distributions (PSDs), which are analyzed using non-local density functional theory (NLDFT) model. The two pores at approximately 1 nm and higher than 1 nm are attributed to slit- and cylindrical-pores between nanoplatelets or nanoparticles.

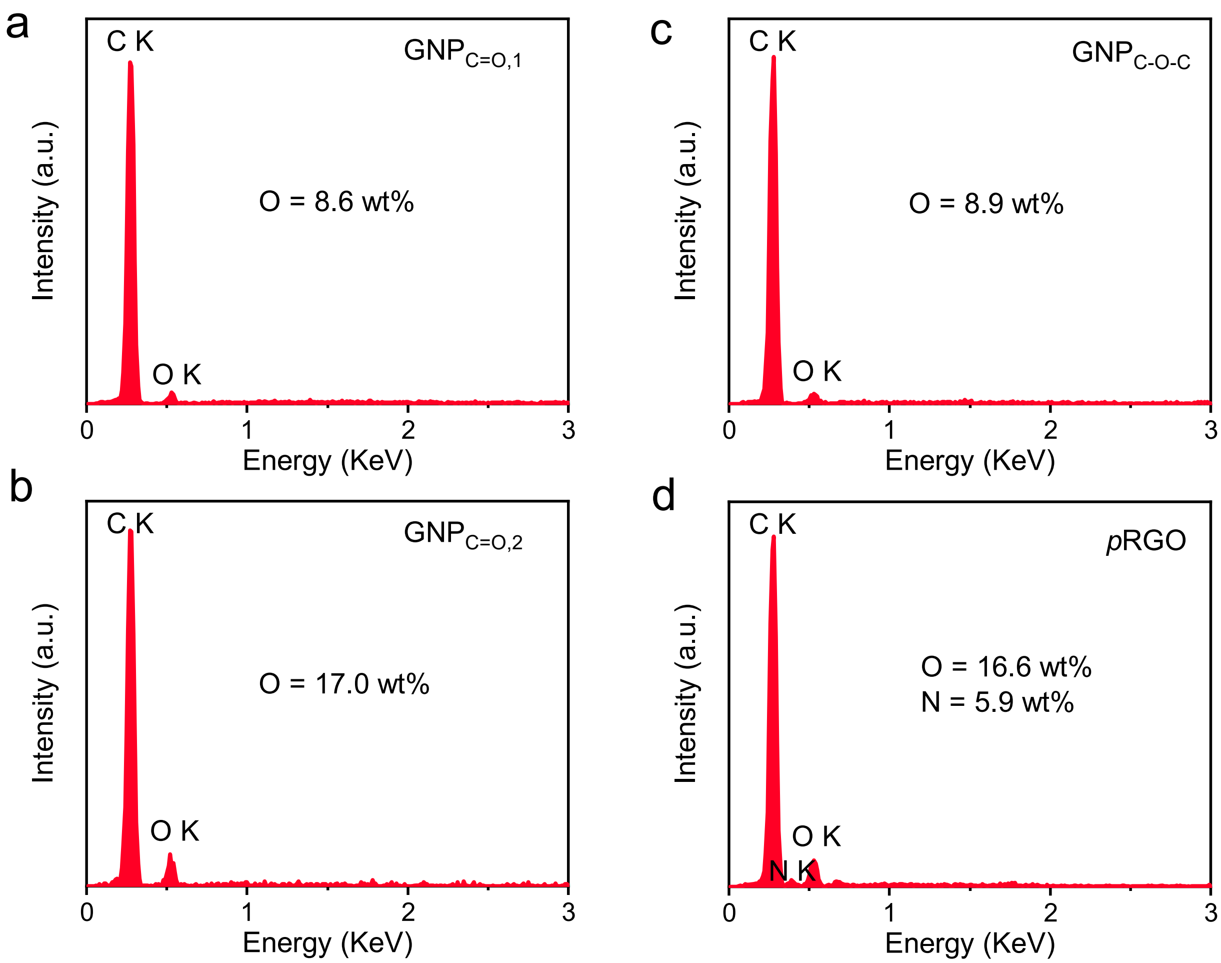

Supplementary Fig. 5 | Energy-dispersive X-Ray spectroscopy (EDS) spectra of samples. **a**, $GNP_{C=O,1}$. **b**, $GNP_{C=O,2}$. **c**, $GNP_{C\text{-}O\text{-}C}$. **d**, *p*RGO.

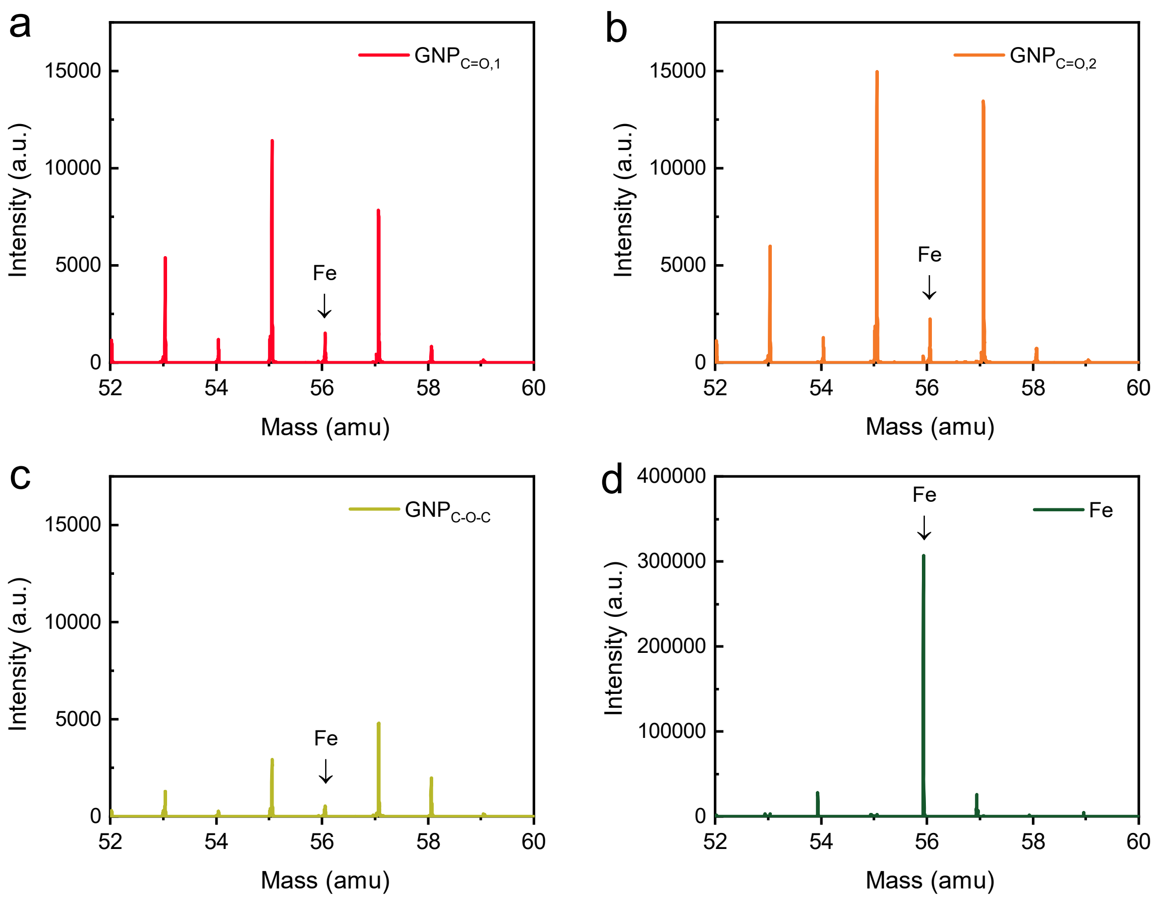

Supplementary Fig. 6 | Time-of-flight secondary ion mass spectrometry (TOF-SIMS) spectra of samples. **a**, $GNP_{C=O,1}$. **b**, $GNP_{C=O,2}$. **c**, $GNP_{C\text{-}O\text{-}C}$. **d**, Fe.

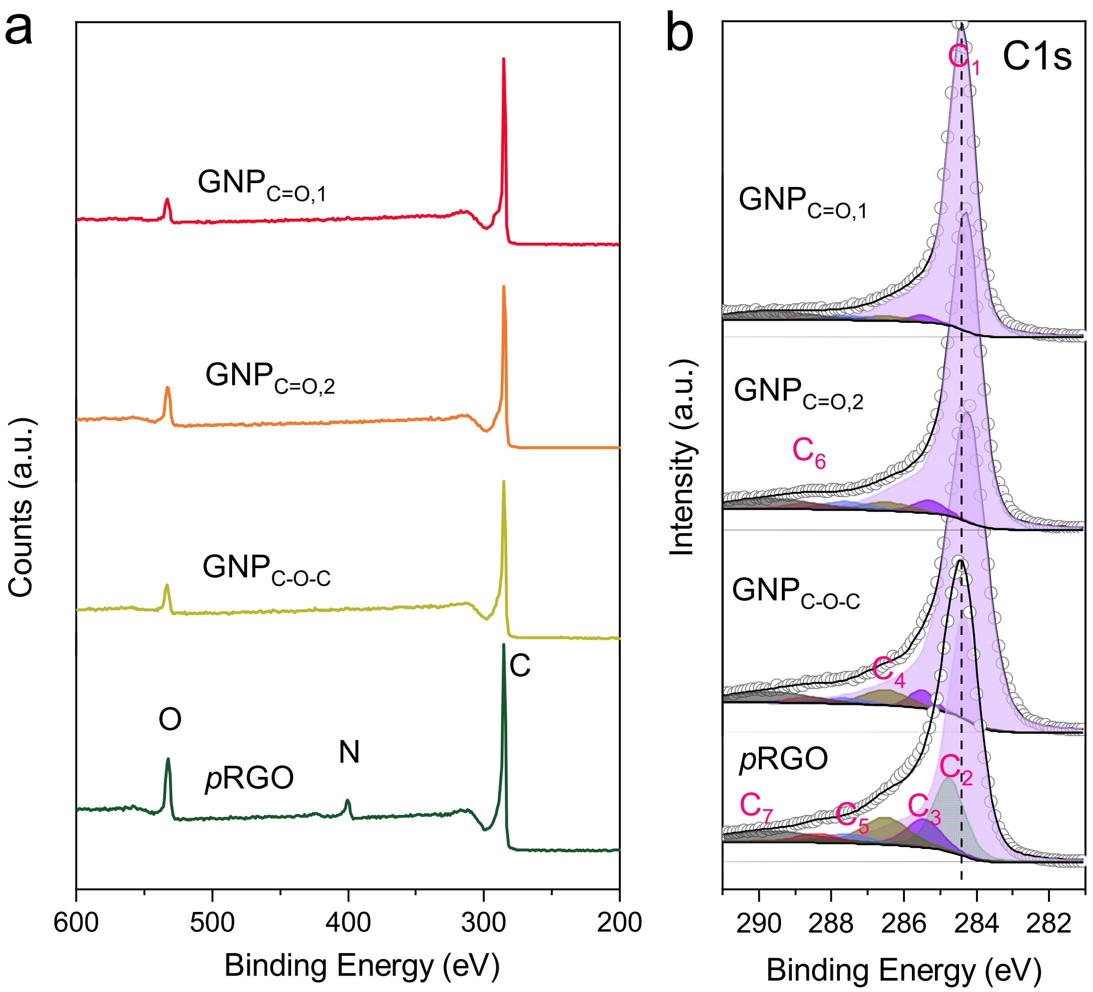

Supplementary Fig. 7 | X-ray photoelectron spectra (XPS) of graphitic nanoplatelets ($GNP_{C=O,1}$, $GNP_{C=O,2}$, and $GNP_{C\text{-}O\text{-}C}$) and custom synthesized partially reduced graphene oxide (*p*RGO). **a**, Full survey spectra. **b**, High-resolution C 1s spectra. C_1, 284.30 ±0.05 eV, *sp*^{2} C-C; C_2, 284.75 eV, aromatic C-H; C_3, 285.4 ±0.1 eV, *sp*^{3} C-C; C_4, 286.5 eV, C-O; C_5, 287.6 eV, C=O; C_6, 288.6 ±0.2 eV, COO; C_7, 289.7 eV, adsorbed H_2O and O_2, as well as π-π*.

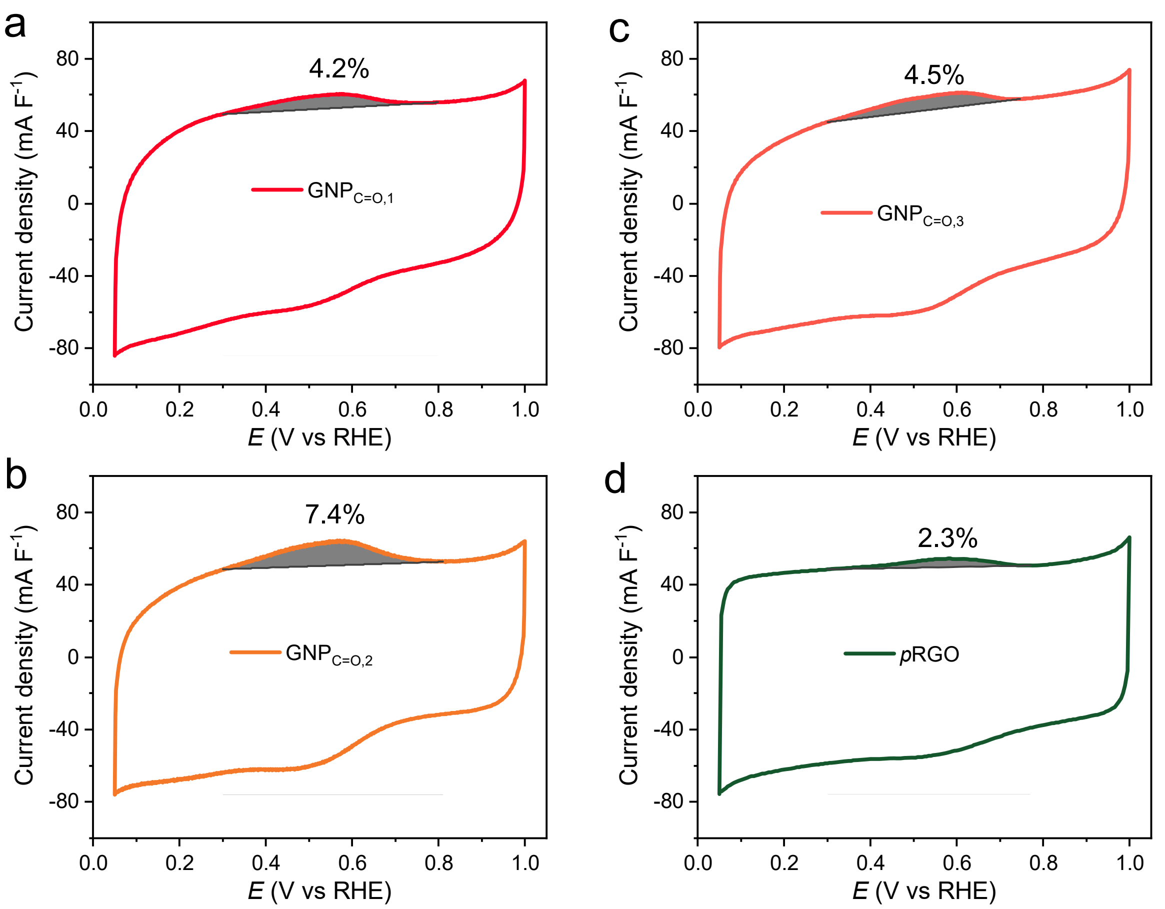

Supplementary Fig. 8 | The calculations of quinone content by CV method. **a**, GNP$_{C=O,1}$. **b**, GNP$_{C=O,2}$. **c**, GNP$_{C=O,3}$. **d**, *p*RGO.

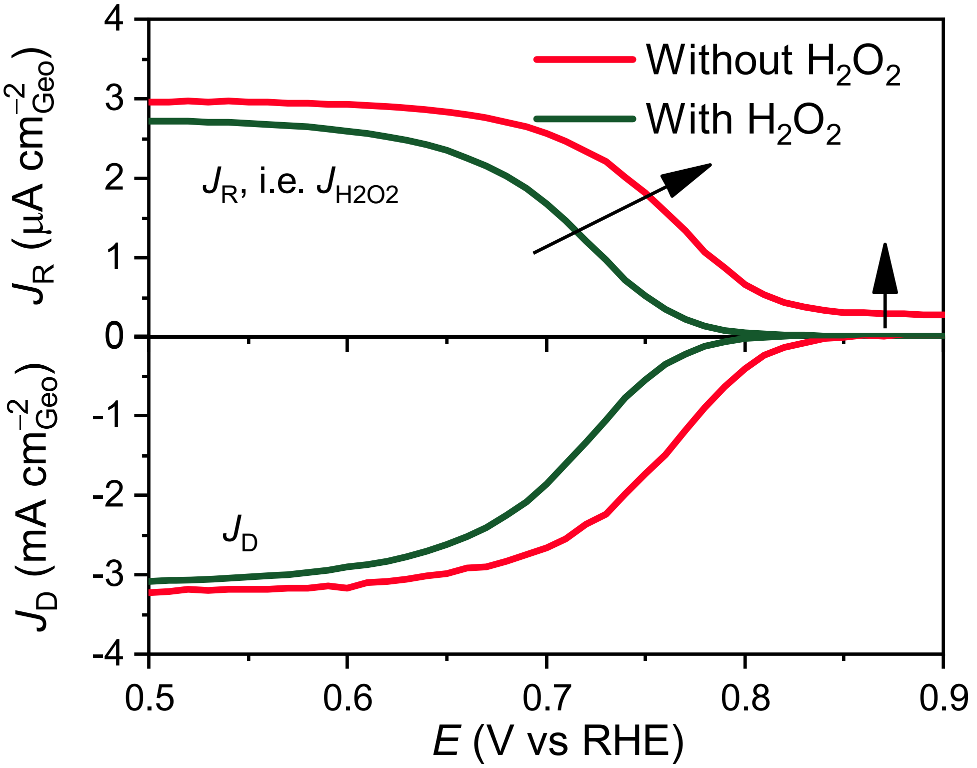

Supplementary Fig. 9 | Potential shift curves due to the presence of H_2O_2. H_2O_2 was *in-situ* generated by using the chronoamperometry method, in which the applied potential was 0.65 V for 2000 s. The rotation speed was 1600 rpm.

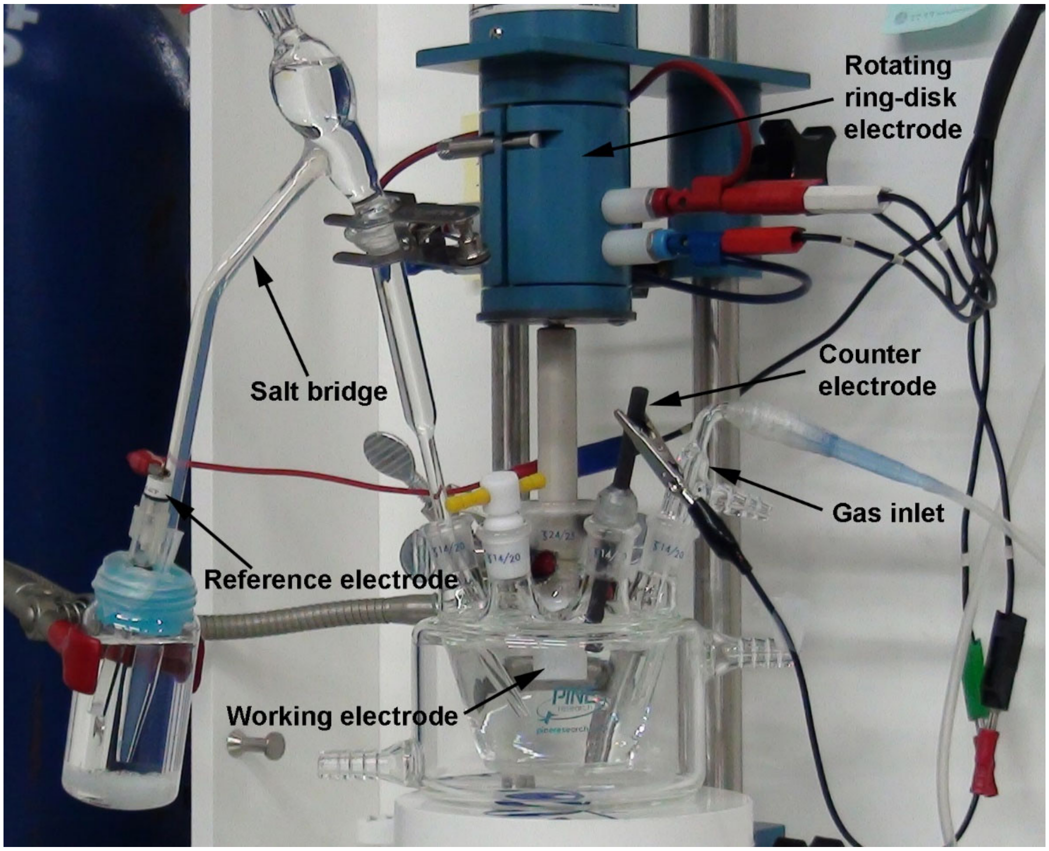

Supplementary Fig. 10 | Rotating ring-disk electrode (RRDE) device equipped with salt bridge. A salt bridge was adopted to connect the electrochemical cell and reference electrode. A salt bridge is used to eliminate a potential shift caused by generated H_2O_2.

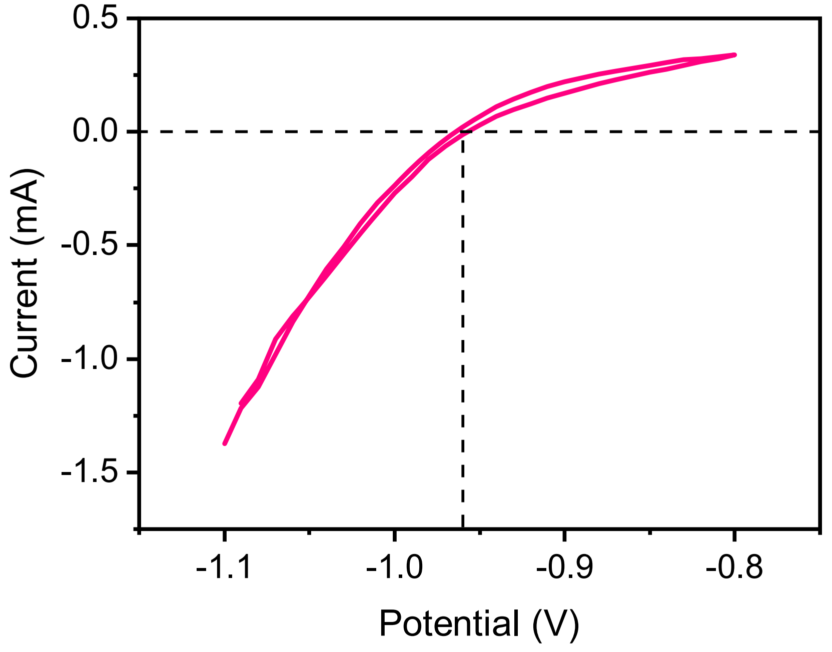

Supplementary Fig. 11 | Potential calibration. The zero potential of the reversible hydrogen electrode (RHE) was obtained on a commercial Pt black coated electrode in an H_2-saturated electrolyte at a scan rate of 1 mV s^{-1}.

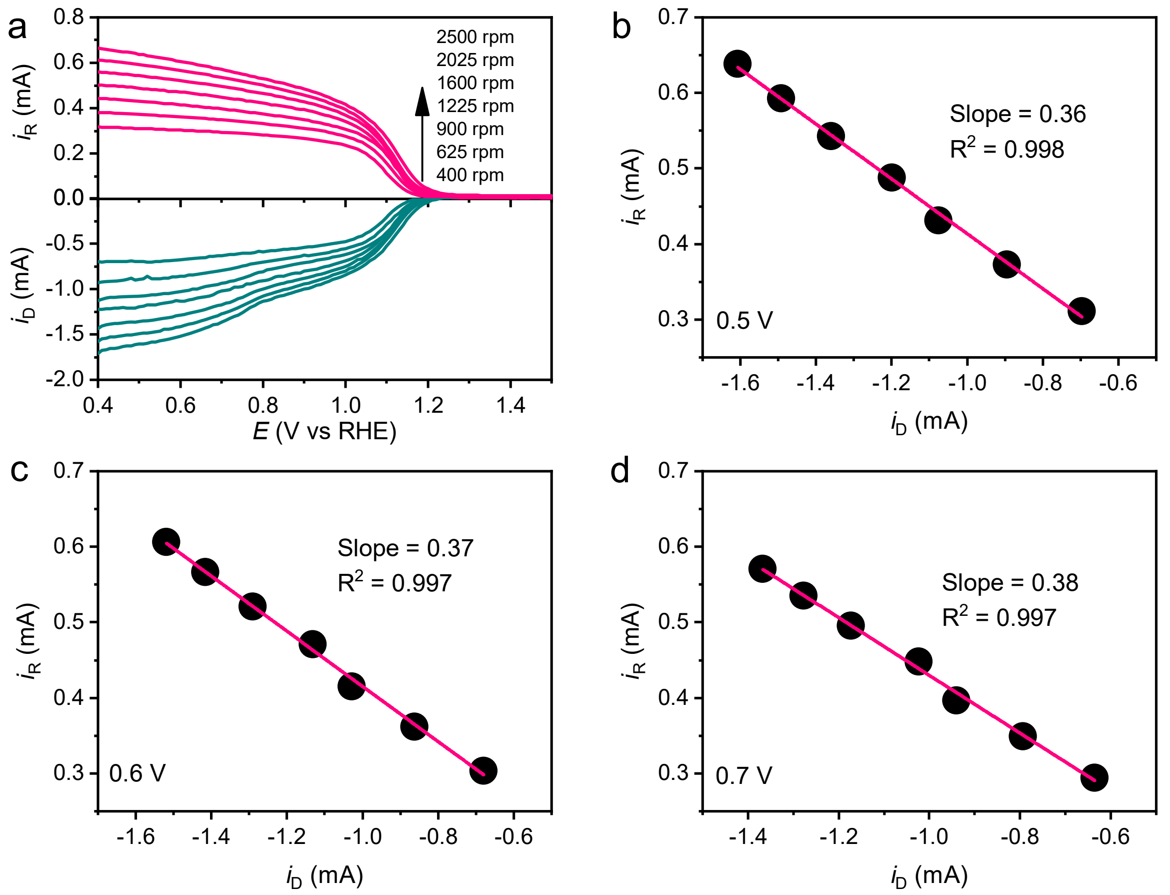

Supplementary Fig. 12 | Determination of the collection efficiency. **a**, Ring (I_R) and disk (I_D) currents for determining collection efficiency on a commercial Pt/C coated RRDE electrode in 0.1 M aq. KOH supporting electrolyte with 10 mM aq. $Na_3Fe(CN)_6$. Scan rate: 20 mV s^{-1}. Applied ring potential: 1.55 V. **b–d**, The fitting results of the determined collection efficiency at potentials of 0.5, 0.6, and 0.7 V, respectively.

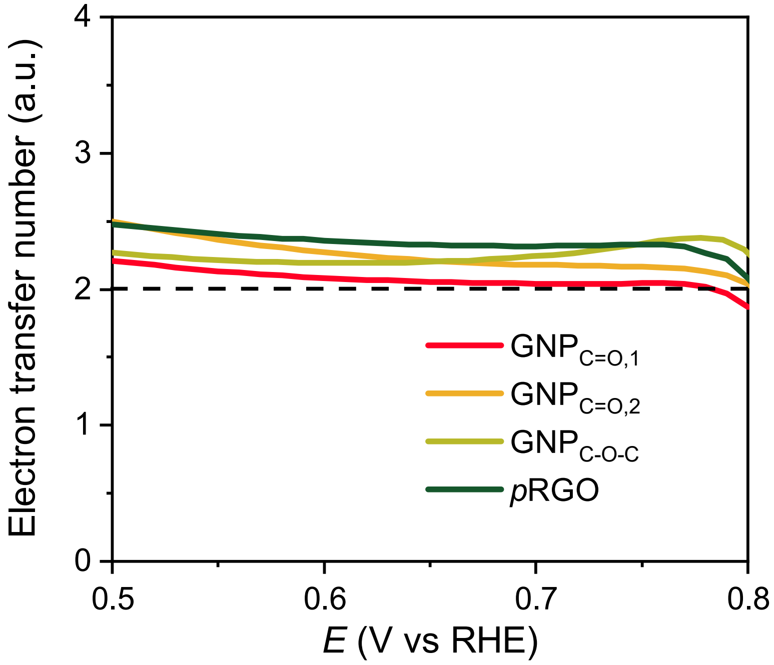

Supplementary Fig. 13 | Performance of oxygen reduction to hydrogen peroxide (ORHP). The plot shows the electron transfer number with respect to applied potential.

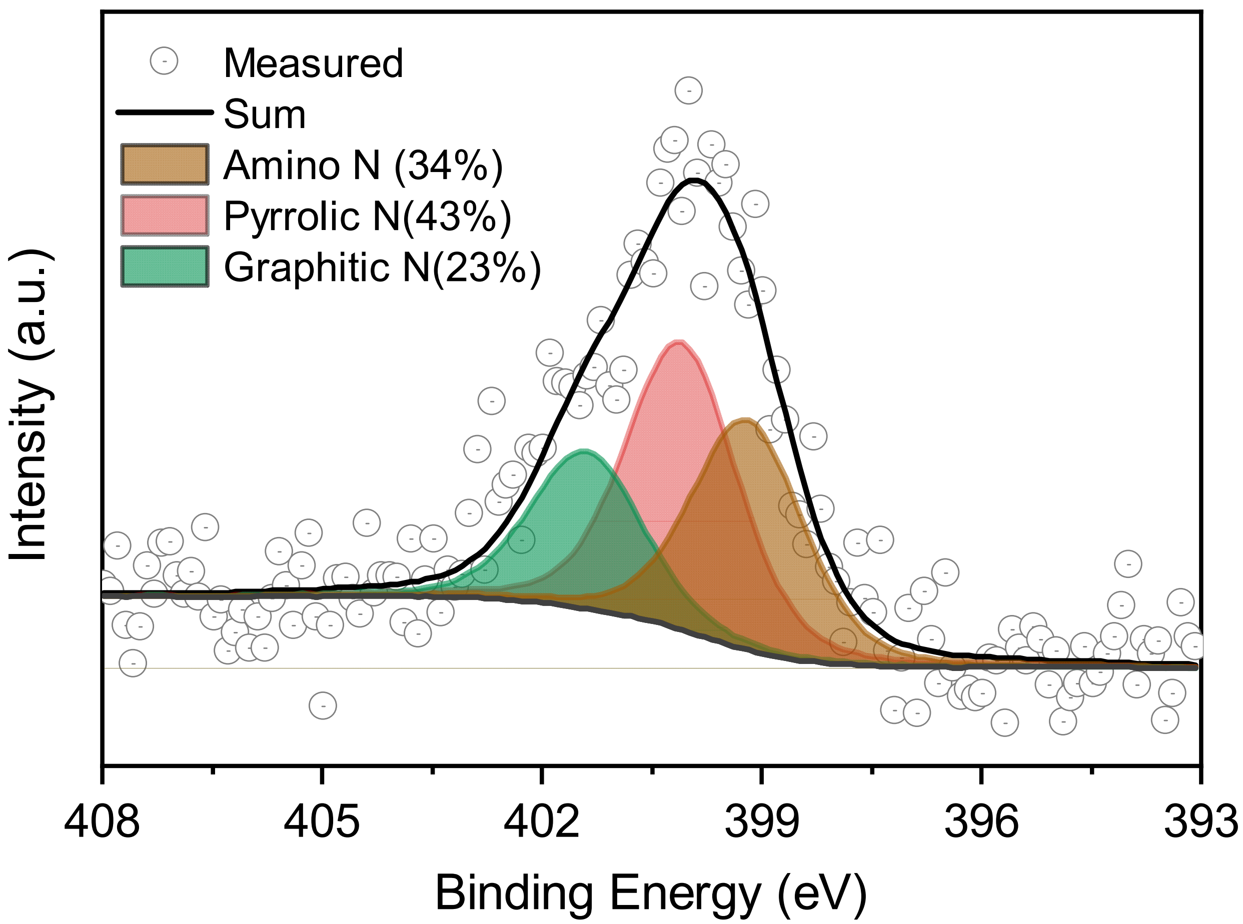

Supplementary Fig. 14 | The high-resolution N 1s of the X-ray photoelectron spectra (XPS) for *p*RGO. Amino N, 399.2 eV, 34%; Pyrrolic N, 400.1 eV, 43%; Graphitic N, 401.4 eV, 23%.

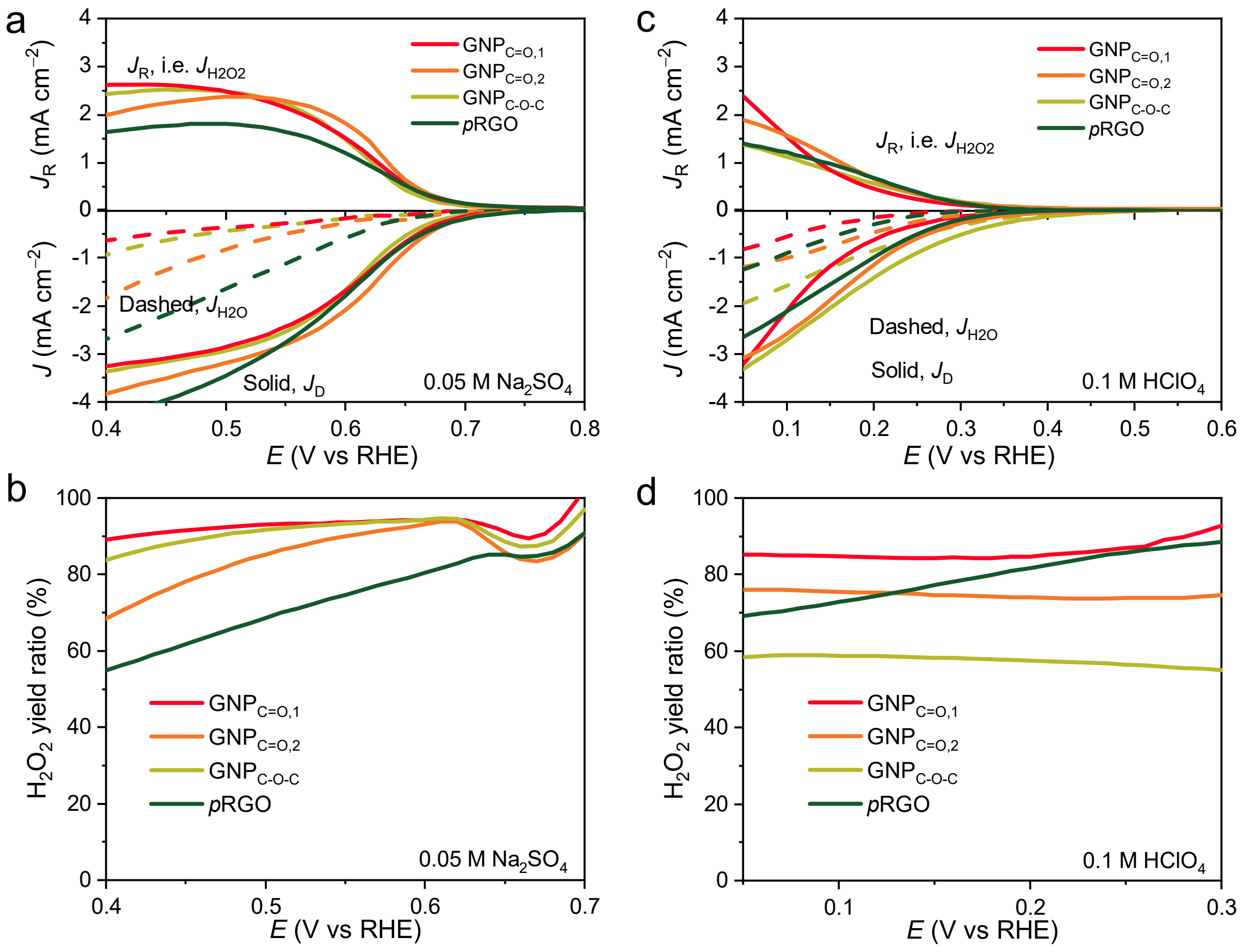

Supplementary Fig. 15 | Performance of oxygen reduction to hydrogen peroxide (ORHP) in neutral and acidic media. a, The polarization curves of H_2O_2 current (J_R or J_{H2O2}), disk current (J_D), and H_2O current (J_{H2O}). The curves were measured in O_2-saturated 0.1 M $HClO_4$ solution at a scan rate of 10 mV s^{-1} by RRDE with a rotation speed of 1600 rpm. The applied potential of the ring was 1.15 V. The current was the average of the forward and backward scans. **b**, The corresponding H_2O_2 yield ratio. **c**, The polarization curves were measured in O_2-saturated 0.05 M Na_2SO_4 solution at a scan rate of 10 mV s^{-1} by RRDE with a rotation speed of 1600 rpm. The applied potential of the ring was 1.15 V. The current was the average of the forward and backward scans. **d**, The corresponding H_2O_2 yield ratio.

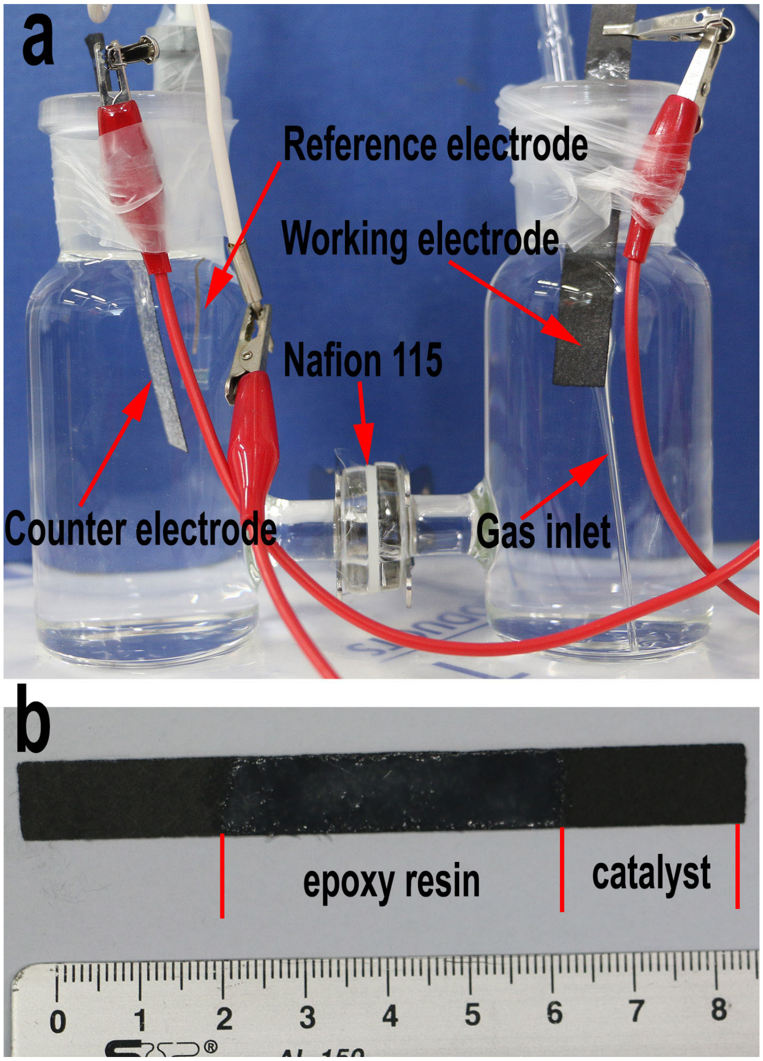

Supplementary Fig. 16 | The stability test device. **a**, H-type cell. The anodic cell and the cathodic cells were separated by Nafion 115 membrane. **b**, Working electrode, which is partially capped with epoxy resin.

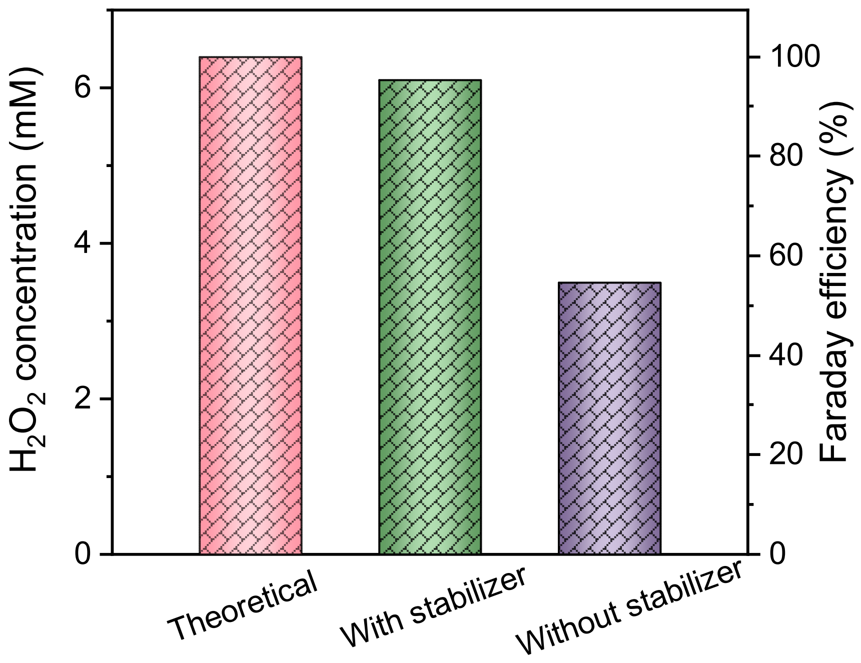

Supplementary Fig. 17 | Faraday efficiency during stability test. The addition of a stabilizer can effectively suppress the decomposition of hydrogen peroxide in a base solution.

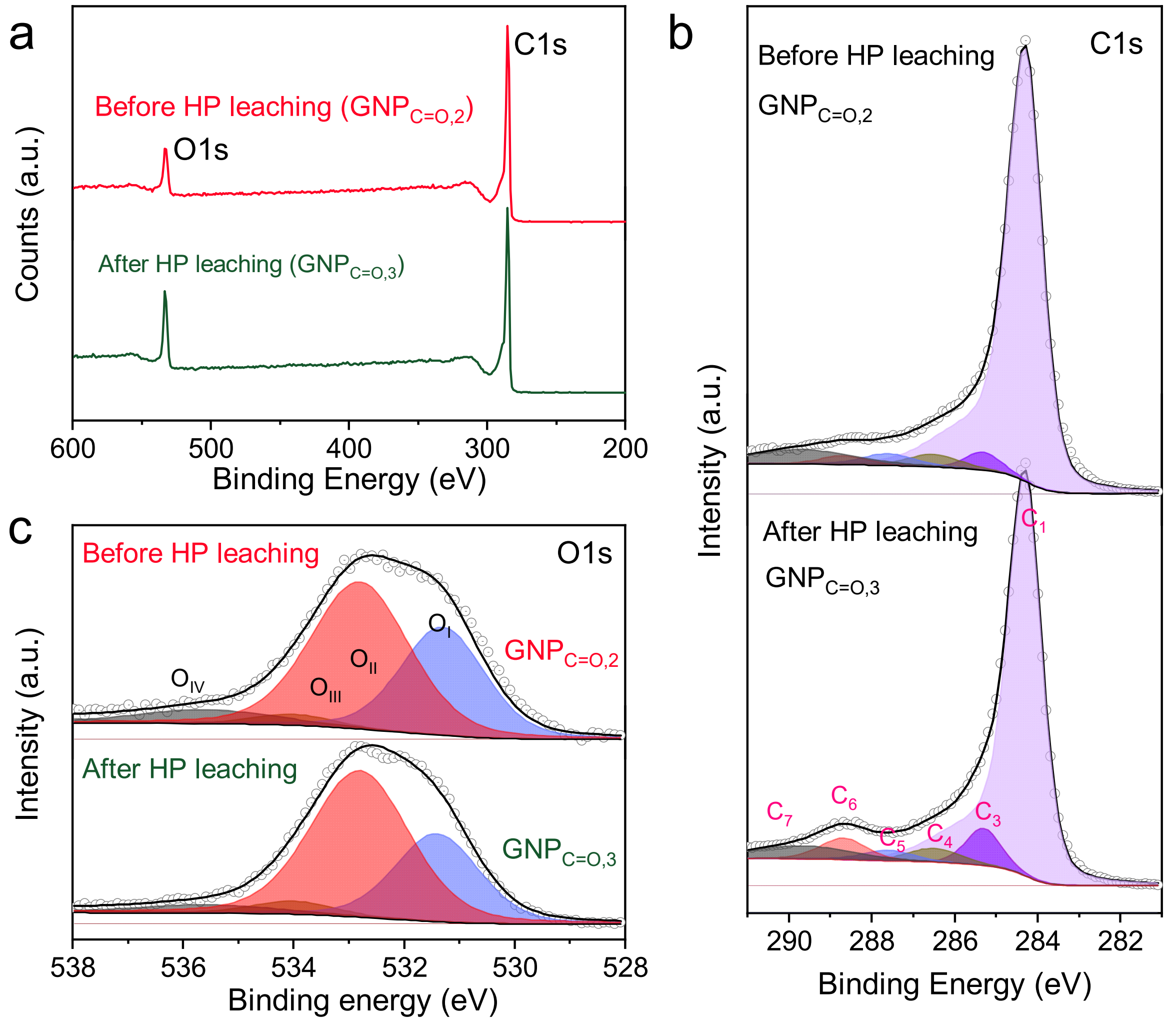

Supplementary Fig. 18 | X-ray photoelectron spectra (XPS) of graphitic nanoplatelets (GNP$_{C=O,2}$) before and after concentrated hydrogen peroxide leaching in acid solution (GNP$_{C=O,3}$). **a**, Full survey spectra. **b**, High-resolution C 1s spectra. C_1, 284.30 ±0.05 eV, *sp*^{2} C-C; C_2, 284.75 eV, aromatic C-H; C_3, 285.4 ±0.1 eV, *sp*^{3} C-C; C_4, 286.5 eV, C-O; C_5, 287.6 eV, C=O; C_6, 288.6 ±0.2 eV, COO; C_7, 289.7 eV, adsorbed H_2O and O_2, as well as π-π*. **c**, High-resolution O 1s spectra. O_I, 531.35±0.05 eV, C=O, quinone, and ketone; O_{II}, 532.8 eV, C-O-C, or COOH; O_{III}, 534.0 eV, C-O(H); O_{IV}, 535.6 eV, adsorbed H_2O and O_2.

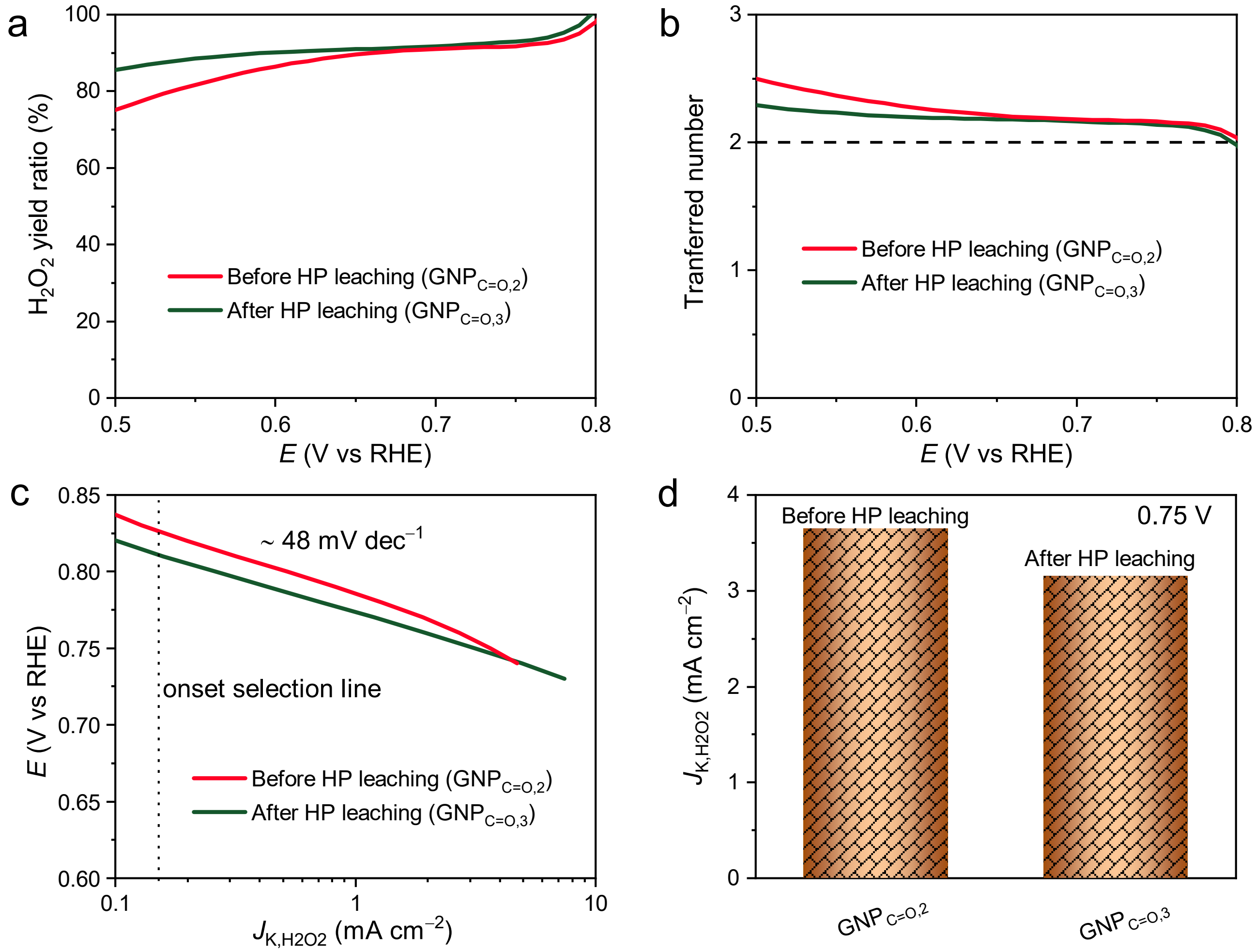

Supplementary Fig. 19 | Determination of active sites by comparing the $GNP_{C=O}$ before ($GNP_{C=O,2}$) and after ($GNP_{C=O,3}$) concentrated hydrogen peroxide leaching in acid solution. **a**, Corresponding H_2O_2 yield ratio. **b**, Electron transfer number. **c**, Tafel plots of $J_{K,H2O2}$. **d**, $J_{K,H2O2}$ at 0.75 V.

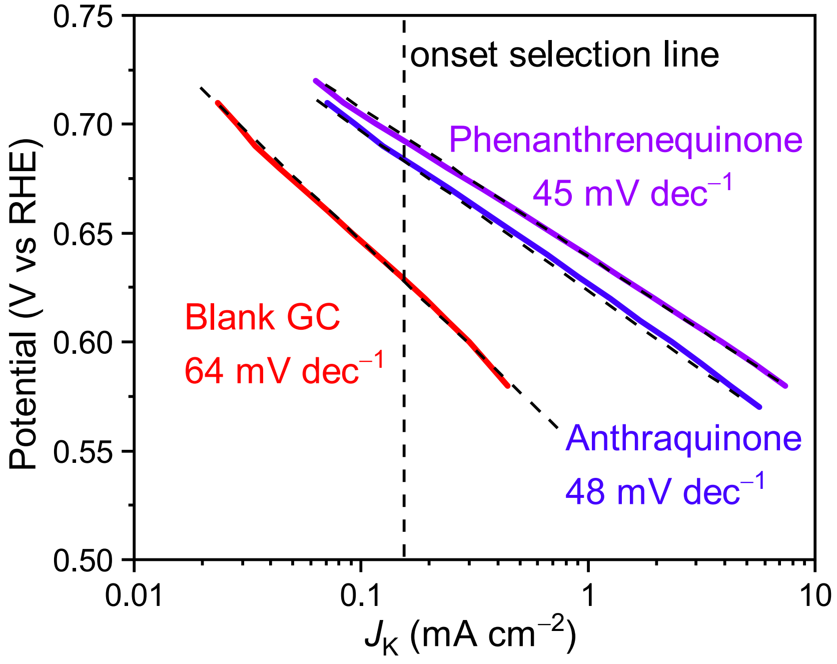

Supplementary Fig. 20 | Performance of oxygen reduction to hydrogen peroxide (ORHP). Tafel plot of phenanthrenequinone, anthraquinone, and the blank glass carbon (GC). The onset potential was defined as the potential measured at a current density of 0.15 mA cm^{-2} (5 % of the theoretical limiting current) for ORHP.

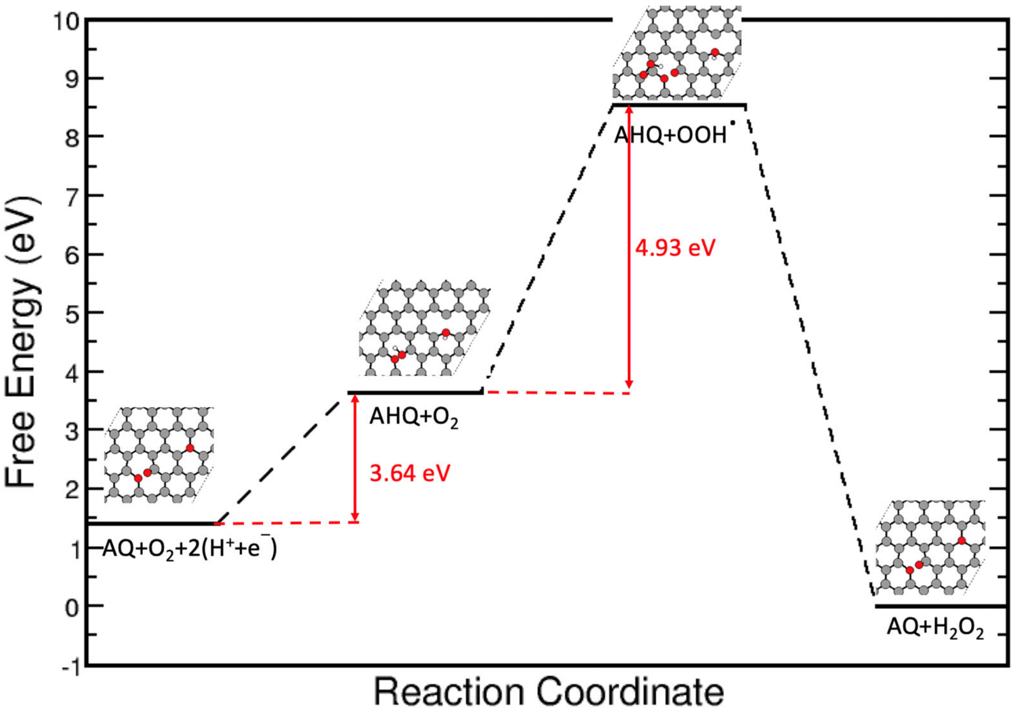

Supplementary Fig. 21 | Calculated reaction free energy diagram for a mechanism, which is similar to the industrial anthraquinone process. AQ and AHQ denote anthraquinone and anthrahydroquinone, respectively.

a) b)

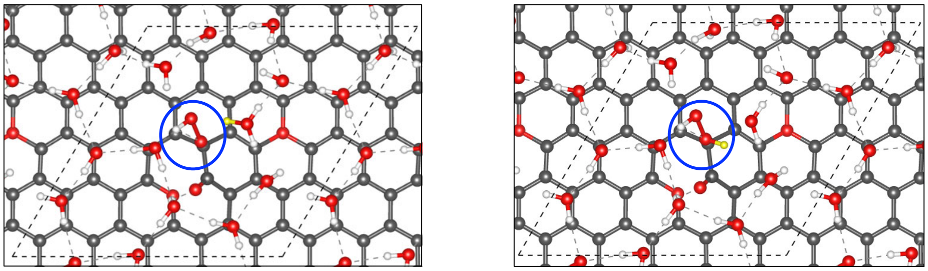

Supplementary Fig. 22 | The initial and final structure models, respectively for calculating proton transfer from hydronium ion to the adsorbed OOH*. a, Initial structure with OOH* adsorbed on graphene surface (circled in blue) and proton in the water structure. **b**, Final structure showing the proton transfer to OOH* adsorbate and forming H_2O_2 product (circled in blue).

Supplementary Tables:

Supplementary Table 1 | Element compositions determined by EA and EDS[a]

Method[b]	EA				EDS	
Element	C (wt%)	H (wt%)	O (wt%)	O / C at/at%	C (wt%)	O (wt%)
$GNP_{C=O,1}$	88.8	0.7	10.5	8.8	91.4	8.6
$GNP_{C=O,2}$	78.0	1.4	20.6	19.9	83.1	17.0
$GNP_{C=O,3}$	71.4	1.5	25.1	26.3	76.5	23.5
GNP_{C-O-C}	89.0	0.3	10.0	8.4	91.1	8.9
*p*RGO[c]	66.6	2.1	21.3	24.0	77.5	16.6

[a] Chemical composition was determined by two methods: element analysis (EA) and energy dispersive spectroscopy (EDS). All data are average values of at least three measurements. The O/C ratios are normalized to atomic percentage of O to C.

[b] Typically, EA results reflect the elemental composition of the bulk sample, while EDS results more likely represent the elemental composition of the sample surface (depending on the penetration depth of the e-beam).

[c] The N contents in the *p*RGO were determined to be 6.4 wt% and 5.9 wt% by EA and EDS, respectively.

Supplementary Table 2 | Statistics of functional group analysis

	Enriched groups	Secondary groups
$GNP_{C=O,1}$	Quinone	Carboxylic acid
$GNP_{C=O,2}$	Carboxylic acid	Quinone
$GNP_{C=O,3}$	Carboxylic acid	Quinone
GNP_{C-O-C}	In-plane etheric ring	Ketone, out-of-plane etheric ring
*p*RGO	Carboxylic acid	Quinone, ether, organic carbonate, aromatic hydrocarbons, phenol

Supplementary Table 3 | Comparison of performance with other reports.

Sample name[a]	$J_{K,H2O2}$ (mA cm^{-2}, 0.65 V)	$J_{K,H2O2}$ (mA cm^{-2}, 0.75 V)	Onset potential (V)[b]	H_2O_2 yield ratio (%, 0.75 V)	Reference
$GNP_{C=O,1}$	25.1	2.7	0.815	97.8	Present work
$GNP_{C=O,2}$	14.9	3.6	0.826	91.7	Present work
GNP_{C-O-C}	11.9	1.6	0.805	83.5	Present work
*p*RGO	6.9	1.2	0.810	83.4	Present work
O-CNTs	10.5	0.4	0.795	90.0	1
BN-C1	6.0	1.5	0.820	88	2
BN-C2	2.1	1.6	0.810	62	2
O-GOMC-8	9.5	0.7	0.800	95.8	3
Co–POC–O	3.5	2.2	0.845	84	4
OXO-G/$NH_3 \cdot H_2O$	1.1	0.2	0.78	79 (0.65 V)	5
Fe-N-C	-	-	-	30 (0.6 V)	6
Cu_3(7-N-Etppz(CH_2OH))	-	-	-	21[c]	7
g-N-CNH	1.8 (0.59 V)	-	0.71	60 (0.65 V)	8
NCMK3IL50_800T	-	-	-	82 (0.3 V)	9
MesoC	2.0	-	0.73	65 (0.65 V)	10
MicroC	0.6	-	0.68	48 (0.65 V)	10
CMK-3	-	2.0	0.795	60	11

[a] All the data were measured in 0.1 M aq. KOH.

[b] The onset potential was defined as the potential, at which the current density of ORHP reached 0.15 mA cm^{-1} (about 5% of the limiting current).

[c] The potential is unknown.

Supplementary Methods:

Preparation of partially reduced graphene oxide (*p*RGO): Graphene oxide was prepared by the Hummers′ method.[12] In detail, the graphene oxide was synthesized by charging purified natural graphite (1 g, Alfa Aesar, 100 mesh, 99.9995 %) and potassium nitrate (0.6 g, Alfa Aesar) in sulfuric acid (25 mL, Sigma Aldrich, 99.999%) in a one-neck round bottom flask (50 mL). The reaction flask was placed in an ice-bath. Then, potassium permanganate (3 g, Sigma Aldrich) was slowly added into the suspension, while vigorously agitating. *Safety Note*: *the temperature of the suspension should be kept lower than 20 °C*. After adding potassium permanganate, the ice-bath was removed and the suspension was held at room temperature for 30 min. Finally, ultra-pure water (50 mL, 18.2 MΩ cm, Direct-Q® 3UV, Millipore Corporation) was slowly added to dilute the suspension, and maintained for 15 min. The suspension was then further diluted with ultra-pure water (150 mL) and treated with diluted hydrogen peroxide (3%, Sigma Aldrich, 30 wt% in H_2O) to remove the residual permanganate and manganese oxides to colorless manganese sulfate.[12] After further careful rinsing with ultra-pure water, the suspension was stored in a refrigerator for subsequent usage.

The chemical conversion of graphite oxide to partially reduced graphene oxide (*p*RGO) was performed by adopting the method in Li *et al.*[13] The typical procedure involved treating a homogeneous suspension (150 mL, approximately 0.6 wt% in H_2O) with ammonia solution (18 mL, Sigma Aldrich, 28–30 wt% in water) and hydrazine monohydrate (2.05 mL, Alfa Aesar, 98 wt%). The calculated weight ratio of hydrazine to GO was around 7:10.[13] After sonication for 20 minutes, the suspension was placed in a water-bath at 95 °C for 1 h.[13] The color of the suspension changed from brown to dark black, which indicated that the reduction had occurred. Finally, after copiously rinsing and freeze-drying in *tert*-butyl alcohol, the resultant RGO was further dried in a vacuum oven at 80 °C for 10 h.

Structural characterization: The soft XANES spectra were recorded with a resolution of 0.2 eV for the K-edge of carbon and oxygen in the total electron yield (TEY) mode. The calibration of photon energy was done using the π* resonance position in the carbon K-edge spectrum of graphite (285.4 eV)[14]. For

the oxygen K-edge, the photon energy was calibrated using the π^* resonance position of carbonate (534 eV) within the high emitting regions[14]. For easy comparison, the spectra were first normalized to the intensity of 280 eV for carbon and 525 eV for oxygen, respectively, and then by the maximum intensity of each spectrum again, so that each spectral line ranged from 0 to 1[15].

Electrochemical measurements: The calibration of the reference electrode potential was performed by measuring the onset potential of the hydrogen oxidation reaction (HOR) for commercial Pt black (Sigma Aldrich). The electrolyte was first bubbled with pure H_2 for 30 min, and a positive scan rate was set to as low as 1 mV s^{-1}. The onset potential of HOR was defined as the point, where the current was bigger than zero. The potential was determined to be −0.980 V vs. Ag/AgCl in 0.1 M aq. KOH solution. The converted potential of RHE (E_{RHE}) equals the measured potential (E_{measured}) subtraction of −0.980 V, namely,

$$E_{\mathrm{RHE}} = E_{\mathrm{measured}} + 0.980\ \mathrm{V} \qquad (1)$$

A typical catalyst ink concentration was 10 mg mL^{-1} in propanol for GNPs and *p*RGO. In order to compare the activity of each sample fairly, the drop-cast working electrode was prepared so that it had the same electrochemical area. Accordingly, the drop-casting volume was not fixed. The typical volume ranged from 3 µl to 15 µl. The resulting current density difference ($i = i_a - i_c$) in the CV curves at 0.9 V was about 800 µA at a scan rate of 50 mV s^{-1}. The loadings of $GNP_{C=O,1}$, $GNP_{C=O,2}$, $GNP_{C=O,3}$, $GNP_{C\text{-}O\text{-}C}$, and *p*RGO were approximately 0.29, 0.22, 0.60, 0.25 and 0.12 mg cm^{-2}, respectively. After drying in N_2 flow, the working electrode was covered with a 5.0 µl 0.1 wt% Nafion solution (Sigma Aldrich).

Unlike other samples, the ink concentrations with the standalone molecules, including phenanthrenequinone, anthraquinone, naphthalenetetracarboxylic dianhydride, perylenetetracarboxylic dianhydride, dibenzodioxin, and dibenzofuran, were 0.1 M mL^{-1} in propanol. The inks (5 µl) with the standalone molecules were drop-cast on the surface of GC electrodes, dried in N_2 flow, and finally covered with a 5.0 µl 0.1 wt% Nafion solution (Sigma Aldrich).

Determination of quinone by CV: The quinone redox was measured by CV method in N_2-saturated 0.5 M aq. H_2SO_4. Here, H_2SO_4 was selected as the supporting electrolyte, because the proton has a much higher mass transfer speed than OH^-, and the Debye length of its electrical double layer (EDL) is smaller than that in a base. The CV curves were recorded at a scan rate of 50 mV s^{-1}. For fair comparison, all CV curves were normalized with the 1 F.

Determination of collection efficiency (*N*): The collection efficiency was determined using the previous report.[16] In brief, commercial Pt/C (20 wt%, Johnson Matthey) was drop-cast on the surface of the disk with a loading of 15 μg_{Pt} cm^{-2} as the working electrode. The electrolyte was Ar-saturated 0.1 M aq. KOH with 10 mM $Na_3Fe(CN)_6$ (ACS reagent, ≥99%, Sigma Aldrich). The disk was scanned at 20 mV s^{-1} between 0.4 to 1.5 V at scan rates of 400, 625, 900, 1225, 1600, 2025, 2500 rpm. The disk current was measured by averaging the forward and backward scans, to mitigate the contribution of capacitance. The disk current was recorded at a constant applied potential of 1.55 V. At this potential, the $[Fe(CN)_6]^{4-}$ that was generated on the disk electrode was oxidized back to $[Fe(CN)_6]^{3-}$ on the ring electrode. The collection efficiency was then determined from the ratio of ring (J_R, i.e. J_{H2O2}) to disk (J_D) current density:

$$N = -\frac{J_R}{J_D} \quad (2)$$

The resulting collection efficiency (*N*) was determined to be 37 ± 1 %, which is consistent with the value (37 %) provided by the manufacturer.

H_2O_2 yield ratio: H_2O_2 yields were obtained using a rotating ring-disk electrode (RRDE) device (MSR, Pine Research Instrumentation, Inc.). The disk electrode was made of glassy carbon with a diameter of 5.61 mm (0.247 cm^2). The Pt ring electrode had an outer diameter of 7.92 mm and an inner diameter of 6.25 mm with a ring-disk gap of 320 μm (Model: AFE7R9GCPT).

Polarization curves were obtained in the O_2-saturated 0.1 M aq. KOH solution at a rotation speed of 1600 RPM. Disk current density (J_D) was collected by CV between 0.05 V and 1.00 V at a scan rate of 10 mV s^{-1}. Ring current density (J_R) was recorded at a constant applied potential of 1.15 V. To rule out the interference of capacitance current, polarization curves were calculated by averaging the forward and backward scans. True activity was calculated by normalizing the current with the capacitance. Here, we did not employ electrochemical specific surface area (ECSA), because it is difficult to find a proper conversion factor. This is due to the presence of pseudocapacitance caused by the functional groups.

Hydrogen peroxide current density (J_{H2O2}) was calculated using the following equation:

$$J_{\mathrm{H2O2}} = \frac{J_{\mathrm{R}}}{N} \tag{3}$$

where N is the collecting efficiency (37 %).

The current density of the byproduct H_2O was determined using the following equation:

$$J_{\mathrm{H2O}} = J_{\mathrm{D}} - J_{\mathrm{H2O2}} \tag{4}$$

The ratio of H_2O_2 yield (H_2O_2 %) was determined from RRDE data, using the following equation:

$$\mathrm{H_2O_2}\ \% = \frac{200 \times J_{\mathrm{H2O2}}}{(J_{\mathrm{D}} + J_{\mathrm{H2O2}})} \tag{5}$$

Stability test in H-type cell: The stability test was conducted in an H-type cell. A 115 Nafion membrane (Sigma Aldrich) separated the anodic and cathodic cells. The counter electrode and reference electrode were placed in the anodic cell, and the working electrode was immersed in the cathodic cell. The electrolyte was 0.1 M aq. KOH. $MgSO_4$ (400 ppm) was added to the cathodic cell as a stabilizer. The working electrode was prepared by drop-casting 40 μL ink (10 mg mL^{-1} with 0.2 wt% Nafion) 4 times on carbon paper (JNT20, JNTG). The typical working area was 1 cm × 2 cm. The other part of the carbon paper was sealed with epoxy resin. Stability was tested using a chronoamperometry method. The applied potential

was 0.65 V. After every 30 h measurement, the electrolyte was replaced with fresh, for a total of four times.

H_2O_2 concentration measurement and the Faraday efficiency: The concentration of yielded H_2O_2 was determined by permanganate titration, which was considered one of the most precise and reliable analytical methods. However, since $KMnO_4$ is partially reduced to MnO_2 in basic solution, which could act as a catalyst for H_2O_2 decomposition, the titration was conducted after acidifying the H_2O_2 solution. Into the 20 mL as-prepared solution, 0.5 M aq. H_2SO_4 (5 mL) was added. The titration reaction follows the following stoichiometry:

$$2\ MnO_4^- + 5\ H_2O_2 + 6\ H^+ \rightarrow 2\ Mn^{2+} + 8\ H_2O + 5\ O_2 \qquad (6)$$

The titration can be observed the purple solution of MnO_4^- is reduced by H_2O_2 into colorless Mn^{2+}. Here, we selected a commercially available potassium permanganate solution (0.02 M, equivalent concentration 0.1 N, Reag. Ph Eur, Sigma Aldrich) as the titration reagent. The molar concentration of H_2O_2 (C_{H2O2}) can be determined by the following equation:

$$C_{H2O2} = 5/2 \times C_{KMnO4} \times V_{KMnO4} / V_{H2O2} \qquad (7)$$

where C_{KMnO4} is the molar concentration of the potassium permanganate solution, which is equal to 0.02 M; V_{KMnO4} is the titration volume of the potassium permanganate solution; the V_{H2O2} is the volume of hydrogen peroxide solution used for analysis.

Faraday efficiency during the stability test was determined using the following equation:

$$\text{Faraday efficiency } \% = \frac{n \times F \times C_{H2O2} \times V_{H2O2}}{\int I\, dt} \qquad (8)$$

where n is the electron transfer number, which is 2 for the dioxygen reduction into H_2O_2; F is the Faraday constant (96485.3 C mol^{-1}); $\int I\, dt$ is the consumed quantity of electric charge (C).

Supplementary References:

1. Lu, Z. *et al.* High-efficiency oxygen reduction to hydrogen peroxide catalysed by oxidized carbon materials. *Nat. Catal.* **1**, 156–162 (2018).

2. Chen, S. *et al.* Designing boron nitride islands in carbon materials for efficient electrochemical synthesis of hydrogen peroxide. *J. Am. Chem. Soc.* **140**, 7851–7859 (2018).

3. Sa, Y. J., Kim, J. H. & Joo, S. H. Active edge-site-rich carbon nanocatalysts with enhanced electron transfer for efficient electrochemical hydrogen peroxide production. *Angew. Chemie Int. Ed.* **58**, 1100–1105 (2019).

4. Li, B., Zhao, C., Liu, J. & Zhang, Q. Electrosynthesis of hydrogen peroxide synergistically catalyzed by atomic Co–N_x–C sites and oxygen functional groups in noble-metal-free electrocatalysts. *Adv. Mater.* **31**, 1808173 (2019).

5. Han, L. *et al.* In-plane carbon lattice-defect regulating electrochemical oxygen reduction to hydrogen peroxide production over nitrogen-doped graphene. *ACS Catal.* **9**, 1283–1288 (2019).

6. Choi, C. H. *et al.* Unraveling the nature of sites active toward hydrogen peroxide reduction in Fe-N-C catalysts. *Angew. Chemie Int. Ed.* **56**, 8809–8812 (2017).

7. Thiyagarajan, N. *et al.* A carbon electrode functionalized by a tricopper cluster complex: Overcoming overpotential and production of hydrogen peroxide in the oxygen reduction reaction. *Angew. Chemie Int. Ed.* **57**, 3612–3616 (2018).

8. Iglesias, D. *et al.* N-doped graphitized carbon nanohorns as a forefront electrocatalyst in highly selective O_2 reduction to H_2O_2. *Chem* **4**, 106–123 (2018).

9. Sun, Y. *et al.* Efficient electrochemical hydrogen peroxide production from molecular oxygen on nitrogen-doped mesoporous carbon catalysts. *ACS Catal.* **8**, 2844–2856 (2018).

10. Chen, S. *et al.* Defective carbon-based materials for the electrochemical synthesis of hydrogen peroxide. *ACS Sustain. Chem. Eng.* **6**, 311–317 (2018).

11. Chen, Z. *et al.* Development of a reactor with carbon catalysts for modular-scale, low-cost electrochemical generation of H_2O_2. *React. Chem. Eng.* **2**, 239–245 (2017).

12. Hummers, W. S. & Offeman, R. E. Preparation of graphitic oxide. *J. Am. Chem. Soc.* **80**, 1339–1339 (1958).

13. Li, D., Müller, M. B., Gilje, S., Kaner, R. B. & Wallace, G. G. Processable aqueous dispersions of graphene nanosheets. *Nat. Nanotechnol.* **3**, 101–105 (2008).

14. Díaz, J., Anders, S., Cossy-Favre, A., Samant, M. & Stöhr, J. Enhanced secondary electron yield from oxidized regions on amorphous carbon films studied by x-ray spectromicroscopy. *J. Vac. Sci. Technol. A Vacuum, Surfaces, Film.* **17**, 2737–2740 (1999).

15. Jeong, H.-K. *et al.* X-ray absorption spectroscopy of graphite oxide. *EPL* **82**, 67004 (2008).

16. Paulus, U. A., Schmidt, T. J., Gasteiger, H. A. & Behm, R. J. Oxygen reduction on a high-surface area Pt/Vulcan carbon catalyst: a thin-film rotating ring-disk electrode study. *J. Electroanal. Chem.* **495**, 134–145 (2001).
