# Supplementary material for: Building and identifying highly active oxygenated groups in carbon materials for oxygen reduction to H2O2
Source: Nat Commun. 2020 May 5;11:2209. doi: 10.1038/s41467-020-15782-z (PMC7200778; doi:10.1038/s41467-020-15782-z)
Supplement: Supplementary file 3 — Description of Additional Supplementary Files [file 41467_2020_15782_MOESM3_ESM.pdf]

Description of Additional Supplementary Files

File Name: Supplementary Movie 1

Description: **H_2O_2 titration with aqueous $KMnO_4$ solution.** The typically resulting H_2O_2 concentration was about 6.1 mM after a 30 h reaction. The concentration of $KMnO_4$ was 0.02 M. See methods for more detailed information.
